# The diversity of small non-coding RNAs in the diatom *Phaeodactylum tricornutum*

**DOI:** 10.1186/1471-2164-15-698

**Published:** 2014-08-20

**Authors:** Alessandra Rogato, Hugues Richard, Alexis Sarazin, Björn Voss, Soizic Cheminant Navarro, Raphaël Champeimont, Lionel Navarro, Alessandra Carbone, Wolfgang R Hess, Angela Falciatore

**Affiliations:** Laboratory of Computational and Quantitative Biology, Sorbonne Universités, UPMC Univ Paris 06, UMR 7238, F-75006 Paris, France; CNRS UMR7238, LCQB, F-75006 Paris, France; Institute of Biosciences and Bioresources, CNR, Naples, Italy; Department of Biology, Swiss Federal Institute of Technology Zürich (ETH-Z), Zürich, Switzerland; Genetics and Experimental Bioinformatics, University of Freiburg, Freiburg, Germany; Institut de Biologie de l’Ecole Normale Supérieure (IBENS), Centre National de la Recherche Scientifique UMR8197, Institut National de la Santé et de la Recherche Médicale, U1024 Paris, France; Institut Universitaire de France, Paris, France

**Keywords:** Diatoms, *Phaeodactylum tricornutum*, Small RNAs, tRNAs, U2 snRNA, Transposable Elements, DNA methylation, Periodic small RNAs distribution

## Abstract

**Background:**

Marine diatoms constitute a major component of eukaryotic phytoplankton and stand at the crossroads of several evolutionary lineages. These microalgae possess peculiar genomic features and novel combinations of genes acquired from bacterial, animal and plant ancestors. Furthermore, they display both DNA methylation and gene silencing activities. Yet, the biogenesis and regulatory function of small RNAs (sRNAs) remain ill defined in diatoms.

**Results:**

Here we report the first comprehensive characterization of the sRNA landscape and its correlation with genomic and epigenomic information in *Phaeodactylum tricornutum*. The majority of sRNAs is 25 to 30 nt-long and maps to repetitive and silenced Transposable Elements marked by DNA methylation. A subset of this population also targets DNA methylated protein-coding genes, suggesting that gene body methylation might be sRNA-driven in diatoms. Remarkably, 25-30 nt sRNAs display a well-defined and unprecedented 180 nt-long periodic distribution at several highly methylated regions that awaits characterization. While canonical miRNAs are not detectable, other 21-25 nt sRNAs of unknown origin are highly expressed. Besides, non-coding RNAs with well-described function, namely tRNAs and U2 snRNA, constitute a major source of 21-25 nt sRNAs and likely play important roles under stressful environmental conditions.

**Conclusions:**

*P. tricornutum* has evolved diversified sRNA pathways, likely implicated in the regulation of largely still uncharacterized genetic and epigenetic processes. These results uncover an unexpected complexity of diatom sRNA population and previously unappreciated features, providing new insights into the diversification of sRNA-based processes in eukaryotes.

**Electronic supplementary material:**

The online version of this article (doi:10.1186/1471-2164-15-698) contains supplementary material, which is available to authorized users.

## Background

Non-protein coding RNAs (ncRNAs) play a critical role in maintaining cellular activity and are engaged in a wide variety of molecular tasks and functions in all studied eukaryotic organisms. For instance, transfer RNAs (tRNAs) and ribosomal RNAs (rRNAs) are essential actors of protein translation, small nuclear RNAs (snRNAs) are involved in the pre-mRNA splicing, nucleolar RNAs (snoRNAs) direct the processing and modification of ribosomal RNAs. In addition, a plethora of other types of ncRNAs of variable size (small RNAs of 20–32 nucleotides up to heterogeneous long ncRNAs of several kilobases) has been reported in different kingdoms of life [1–7]. Although some of these ncRNA species show overlapping features in plants and animals, the information concerning their evolution and functionality is still limited [8]. Until now, the most extensively characterized endogenous ncRNAs are short interfering RNAs (siRNAs), also referred to as endo-siRNAs, and microRNAs (miRNAs), which are generated by the processing of double stranded RNAs (dsRNAs) by a Dicer-class RNase III enzyme [1, 9, 10]. These small RNAs are loaded into RNA-induced silencing complex (RISC) containing Argonaute (AGO) proteins, and guide these protein complexes onto sequence complementary mRNA targets to direct their silencing at the post-transcriptional level through mRNA degradation and/or translation inhibition. Small RNAs, and particularly siRNAs, can also guide AGO-RISC onto sequence complementary DNA targets to direct cytosine DNA methylation and/or chromatin modifications resulting in their transcriptional gene silencing [11]. Besides these small RNA species, a variety of new small RNA classes, involved in novel biological functions such as DNA elimination and DNA repair [12–15], continues to be discovered in different organisms. A class of animal small RNA species that has been recently extensively characterized are PIWI-interacting small RNAs (piRNAs) [16]. These DICER-independent small RNA species are 24-32 nt in length and are derived from long precursor transcripts that originate from distinct genomic regions referred to as piRNA clusters. Mature piRNAs bind specifically to the PIWI subfamily of AGO proteins to promote silencing of transposable elements (TEs) -but also of some protein-coding genes- at the transcriptional and post-transcriptional levels [16]. In mice, this pathway has been mostly characterized in the male germ line, where piRNAs presumably direct transcriptional silencing of TEs through DNA methylation [17, 18], a mechanism that is thus analogous to RNA-directed DNA methylation (RdDM) initially described in plants [19].

The existence of a sophisticated level of regulation of gene expression involving interactions and cross-talk between different small RNAs, generated by different RNA silencing pathways, becomes more and more apparent [1]. Furthermore, several recent discoveries (e.g., the identification of snoRNAs with microRNA-like function [20], the finding of small RNAs derived from tRNAs (tRFs) [12], the co-regulation of miRNA biogenesis and splicing from the same host mRNA [21]) shed light on even more complex and unanticipated RNA regulatory networks involving various ncRNA populations with different origins and functions.

Marine diatoms represent a valuable biological system to address questions about the evolution and diversification of gene regulatory mechanisms in eukaryotes. This hugely diverse phytoplanktonic group of the Stramenopiles derives from a secondary endosymbiosis event and shows a peculiar genetic makeup [22, 23]. Most significantly, almost half of the diatom proteins have similar alignment scores to their closest homologs in plants and animals and at least 5% of their gene repertoire has been recruited by horizontal gene transfer from bacteria [23, 24]. As a consequence, diatoms possess a unique mix of bacterial, animal and plant metabolic features. About half of the diatom genes encodes for proteins of unknown function, suggesting that many regulators of central processes in diatom biology remain to be identified. Nevertheless, the ecological success of diatoms in the contemporary oceans and their capability to grow in very different environments imply that these organisms evolved sophisticated mechanisms to efficiently regulate genome structure and expression, mechanisms that still await characterization. Comparative analyses of the diatom genomes have indicated transposable elements as key contributing factors to genome diversification [23, 25]. Moreover, the first genome-wide DNA methylation map in the model diatom *Phaeodactylum tricornutum* has revealed the potential impact of DNA cytosine methylation for the control of transcriptional or post-transcriptional silencing and of differential gene expression under specific growth conditions. The data indicated that around 6% of the genome displays detectable DNA methylation, with extensive methylation being localized over transposable elements [26]. A few studies have also provided an initial characterization of small RNAs in the marine diatoms *Thalassiosira pseudonana* and *P. tricornutum*[27–29]. Putative miRNAs have been predicted in these two species, but none of them has so far been experimentally validated. Broadly speaking, the potential regulatory role of small RNAs in diatoms still awaits to be demonstrated. Interestingly enough, we have shown that the expression of anti-sense or inverted repeat sequences of selected target genes can trigger efficient gene silencing in *P. tricornutum*[30], a clear indication for the existence of RNA interference-like processes in diatoms. Moreover, we have also described the presence of genes encoding a predicted Dicer-like protein, an Argonaute-like protein and a potential RNA-dependent RNA polymerase (RdRP) in the *P. tricornutum* genome [30]. RNAi-related proteins have been identified in *T. pseudonana* as well [27]. Although distantly related to plant or animal RNAi machinery components, these diatom proteins possess conserved structural and functional domains including key amino acid residues that support their potential role in the small RNA pathways described above.

To gain insight into the complexity of small ncRNA biogenesis and function in diatoms, we combined computational and experimental analyses to generate a comprehensive characterization of *P. tricornutum* small RNA populations. In particular, we carried out a high-throughput sequencing of short RNA fragments extracted from cells grown under different conditions of light and iron, two abiotic factors highly relevant for diatom growth in the oceans [31–33]. The characterization of the sRNA transcriptomes revealed a small ncRNA landscape in diatoms that is much more complex than anticipated. We identified and characterized different functional categories of small RNAs with different sizes, suggesting the presence of distinct biosynthetic pathways. Our study also questioned the occurrence of miRNAs, which were not detected. Instead, it identified highly expressed 21-25 nt small RNAs derived from longer non-coding RNAs such as tRNAs and U2 snRNA, or of unknown origin. The majority of small RNAs correspond to TEs, in particular to the Copia-like diatom-specific retrotransposon family [23, 25]. Finally, a clear correlation between the presence of small RNA/DNA methylation and the silencing of TEs and coding genes was established, suggesting that a small RNA-dependent DNA methylation pathway is operational in diatoms as in other plant and animal organisms. An unprecedented periodic pattern of small RNAs on TEs and coding genes, related to the methylation, is also unveiled by our genome-wide analysis, opening a completely novel line of investigation on the molecular determinants establishing sRNA distribution.

## Results

### Genome-wide distribution and length distribution of small RNA populations

To characterize the population of *P. tricornutum* small RNAs (sRNAs), we generated five small RNA libraries within the 18 to 36 nt RNA fraction extracted from cells grown under different conditions: Normal Light (NL), High Light (HL), Low Light (LL), Dark (D), and Iron starvation (−Fe). Their sequencing yielded a total of 8,103,030 reads, which were aligned to the *P. tricornutum* genome [23] and quality filtered, resulting in 7,235,310 reads (89%) aligned to at least one genomic location (Table 1). While the conditions we selected were chosen to represent environmental conditions relevant for photosynthetic growth, the agreement of the sequence pool between any two conditions is high (average Pearson correlation of 0.86 on counts per aligned position). We thus decided to focus on sRNAs that were recovered across multiple conditions.

**Table 1:** **Details of the reads aligned to the** ***P. tricornutum*** **genome**

Condition	Dark (D)	Low light (LL)	Normal light (NL)	High light (HL)	Iron starv. (-Fe)	Total
Reads sequenced	1,926,421	2,053,284	1,601,446	826,528	1,695,351	8,103,030
Aligned (fraction)	1,732,515 (0.9)	1,861,776 (0.91)	1,346,817 (0.84)	744,044 (0.9)	1,550,158 (0.91)	7,235,310 (0.89)
Overlap						
To genes (%)	217,319 (0.13)	233,246 (0.13)	190,670 (0.14)	96,151 (0.13)	167,536 (0.11)	904,922 (0.13)
Repeat regions (%)	821,939 (0.47)	1,039,657 (0.56)	683,533 (0.51)	353,056 (0.47)	643,986 (0.42)	3,542,171 (0.49)
Intergenic regions (%)	362,555 (0.21)	339,074 (0.18)	244,827 (0.18)	116,795 (0.16)	182,682 (0.12)	1,245,933 (0.17)
tRNA genes	36854	51959	40617	46477	87992	263899
Other ncRNAs (RFAM)	89658	34182	47847	28000	126244	325931

For each condition, the summary of the number of reads aligning to each genomic location is reported.

**Figure 1:**
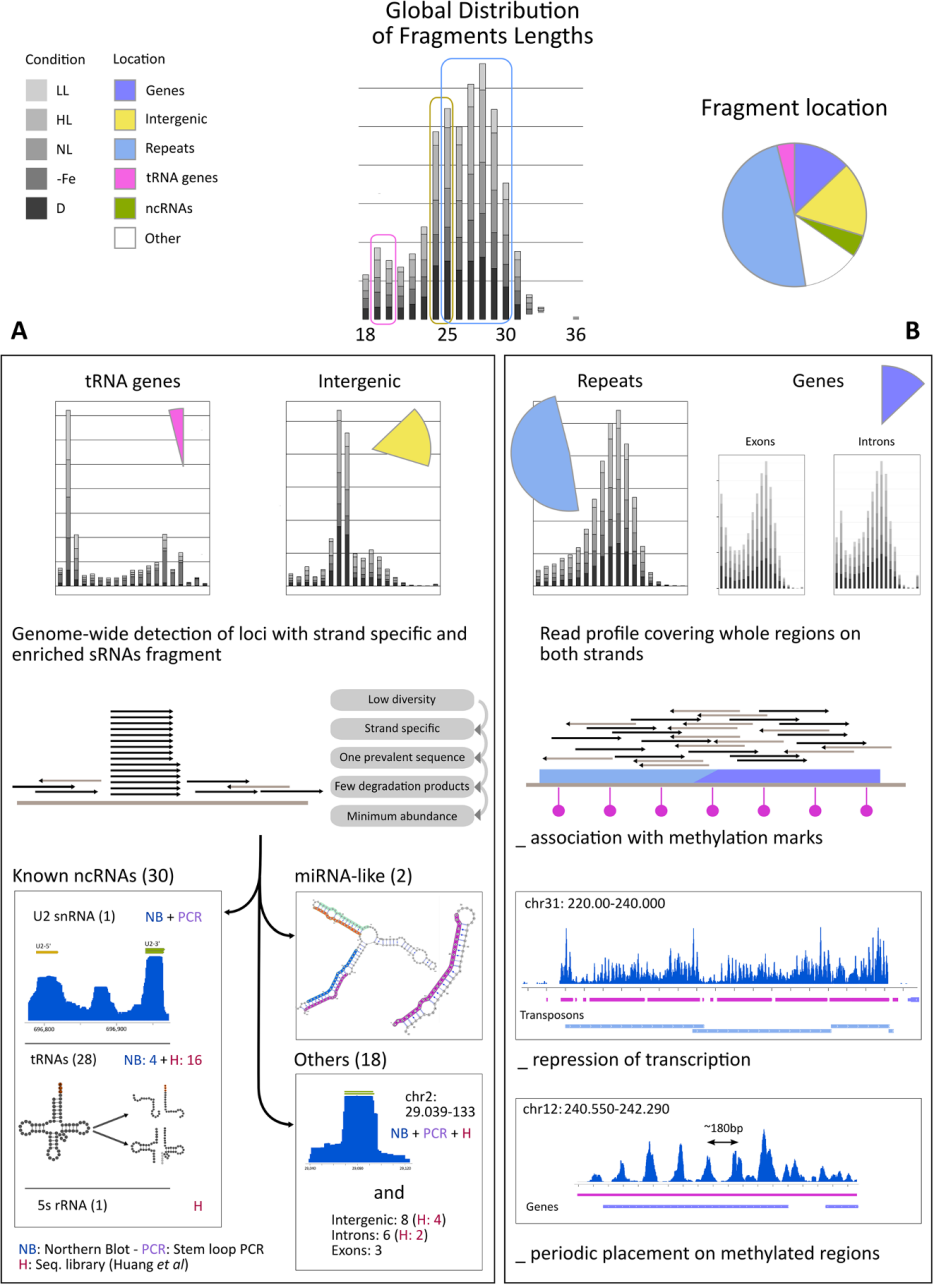
**Workflow of the small RNAs analysis in** ***P. tricornutum.*** **Top.** Fragment lengths distribution of reads (histogram, center) is reported in a grey color scale distinguishing the five experimental conditions (LL, HL, NL, −Fe, D). The distribution of fragment location is also reported (pie chart, right) with a color scale indicating genes, intergenic regions, repeat regions, tRNA genes, ncRNAs and other loci. We distinguish two workflows described in boxes A and B, characterized by different local loci distributions of reads along the genome. **(A)** Sequence specific distribution of fragment lengths that is systematically observed for tRNA genes and intergenic regions. Reads were filtered in five steps, described in the 5 grey boxes. We obtained three main groups of results, indicated by squared boxes (number of predicted sRNAs is reported in parenthesis). The number of predicted sRNAs that were experimentally validated is also indicated, together with the experimental technique (NB, Northern Blot; PCR, Stem Loop PCR; H, sequencing data from [28]). **(B)** Distribution of fragment lengths that covers loci with overlapping reads and accumulated on both strands. This distribution pattern has been observed to either TEs or coding genes, associated to methylation. Examples of the periodic placement of sRNAs on three Codi LTR-retrotransposons on chromosome 31 and on a protein coding gene on chromosome 12 are reported. Color palette for TEs and genes is the same as above, and Highly Methylated regions are represented in purple.

Almost half of the aligned reads (49%) overlaps with repeat regions, followed by a significant fraction located on non-repeat intergenic regions (17%) and coding regions (13%). Notably, 8% of the reads overlap with annotated ncRNAs (Table 1 and Figure 1).

The size distribution of the aligned reads is enriched in three specific lengths, suggesting that distinct sRNA biosynthetic pathways generate the *P. tricornutum* sRNA populations. The three characteristic lengths mainly correspond to tRNA genes, intergenic regions and repeat elements (Figure 1, top). By assessing the distribution on these 3 types of annotations, we observed: 1. a very abundant RNA population of 24-25 nt in length originating from intergenic regions and, in particular, from a unique 80 bp region on chromosome 2 (see below); 2. tRNA fragments mainly enriched in the 19-20 nt fraction and, to a minor extent, in the 30-33 nt fraction; 3. repeat and coding regions enriched in 25-30 nt fragments (see below TE and methylation section).

### An unbiased search for sRNAs reveals diverse classes of novel sRNAs in *P. tricornutum*

Guided by the three populations that have been highlighted by the analysis of read sizes, we explored the landscape of sRNAs with respect to two kinds of sRNA distribution (see workflow description in Figure 1 and Additional file 1: Table S1). Namely, we aligned reads on the *P. tricornutum* genome and observed two behaviors. On the one hand, some reads match very specific genome locations and form, after alignment, characteristic piles of thousands of copies of the same sequence, accumulated on a single strand. On the other hand, there are regions, of a few thousand bases in length, that are covered by overlapping reads, accumulated on both strands and, at times, forming several piles distributed with a periodic pattern (see section on transposons).

For the first distribution type (Figure 1A), after a filtering step eliminating low complexity sequences, strand-specific sequences, potential degradation products and other sequences possibly leading to erroneous sRNA prediction (see Methods) [34, 35], we characterized 50 candidate regions that appeared to be specific to sRNAs lying in tRNAs and in intergenic regions. These are organized in three groups. Thirty regions correspond to already annotated non-coding RNA structures, including an highly abundant candidate (representing 0.4% of all aligned reads) that overlaps the U2 snRNA gene located on chromosome 5, and the 5S ribosomal RNA on chromosome 3, as previously noted for human [36]. The majority of the candidate regions overlap with 28 different tRNA sequences (corresponding to 22 different codons), suggesting that they may represent tRNA-related small RNAs (tRFs), similarly to observations made in a few other organisms [37, 38]. The remaining 20 candidates (Additional file 2: Table S2) do not overlap with regions related to non coding RNAs. Two of them resemble miRNA-like molecules, supported by a stable precursor structure, and 18 of them have no particular associated structure. These predictions were well supported across different libraries (28 regions detected in at least two libraries) and on an independent sequencing experiment published by Huang and coworkers [28] that we integrated in our pipeline (24 regions are also detected).

The second kind of sRNAs distribution appeared to be specific to transposable elements and to coding genes (Figure 1B). Three major observations were made: 1. reads overlap over relatively long regions that are typically methylated, 2. the sRNAs accumulation correlates with repression of transcription, and 3. the sRNA profile displays periodic patterns at a distance varying within the 180-200 nt interval.

This analysis highlights that 93.5% of sequence specific reads can be explained by their accumulation in well classified loci covering 8% of the whole genome (Additional file 1: Table S1 reports precise values found in normal light, but the same holds for different environmental conditions). Several of the sRNAs found in our analysis have been selected for experimental validation (Figure 1 and below).

### The annotation of processed reads yields two miRNA-like sequences

By combining sequencing data and precursor structure prediction, we predicted only two putative miRNA sequences (see Additional file 3: Figure S1 and Additional file 4: Figure S2 for the predicted structures). To do so, we first assessed the existence of a significantly stable precursor secondary structure (see Methods) around an abundant fragment covered by reads, and then verified that this putative miRNA locus was indeed located over a stem together with a proper star sequence, also represented by read accumulation (Methods and Additional file 3: Figure S1 and Additional file 4: Figure S2).

The most represented putative miRNA locus on chomosome 10 (chr10: 42.6924-42.7420) shows a read profile strikingly consistent with the expectations. Precisely, the precursor sequence is predicted to fold in 3 stems (Additional file 3: Figure S1), suggesting the presence of a polycistronic miRNA cluster as previously reported in other organisms [39]. The first stem contains the main miRNA locus, a sequence that is expressed in all five experimental conditions, and the second stem shows another putative miRNA locus, only expressed under iron starvation. It is noteworthy that both of these putative miRNAs occur together with a star sequence. However, they show 2 nucleotide 3′ overhangs only in one side, which is not compatible with a Dicer processing that normally result in RNA duplexes with 2 nucleotides 3′ overhangs at both 3′ ends [40], and therefore we named them miRNA-like. A second miRNA-like locus on chromosome 1 (chr1: 2.453.906-2.454.049) is highly overrepresented in all experimental conditions and is supported by a hairpin shaped precursor structure (Additional file 4: Figure S2). However, the read accumulation did not allow the identification of a precise star sequence position. We carried out Northern blot analysis of the identified miRNA-like sequences, but we were unable to detect their expression (data not shown).

In conclusion, although we have predicted two over-represented sequences that may correspond to putative miRNA genes, the general evidence is relatively weak for this class of non-coding RNA, being outnumbered by tRFs (see below) or having only a mild support from RNA structure predictions. Our other attempts to identify *bona fide* miRNAs from genome-wide analyses were unsuccessful: would it be predictions from sequencing data (MIReNA was used in sequencing data mode [29]), or sequences alignment to miRBase v.17 [41]. In the latter case, only 0.02% of the sequences could be aligned and they were distributed across a highly diverse set of mature sequences (data not shown). Moreover, none of the miRNAs previously identified in *P. tricornotum*[28] could be confirmed by our predictions (Additional file 5: Supplementary data). Altogether these results suggest that there might be only very few miRNAs in *P. tricornutum*, and none of them possess known homologues in other species.

### Small RNAs localized in intergenic regions

**Figure 2:**
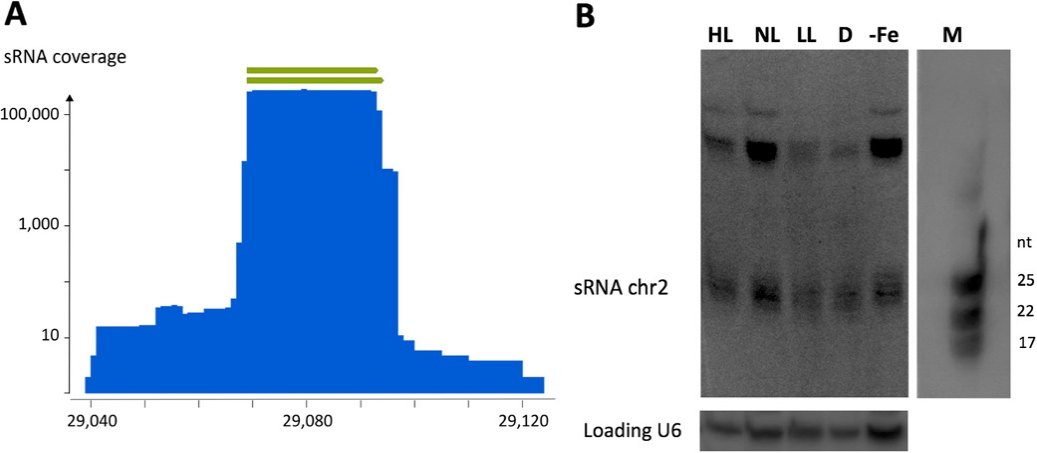
**A novel sRNA population of unknown biogenesis from an intergenic region. (A)** Screenshot of the reads profile of the mature small RNA populations matching an intergenic region in the chromosome 2 (chr2: 29.039-29.123), with reads belonging to the five small RNA libraries. **(B)** Validation of the sRNAs expression by low molecular weight Northern blotting. Total RNA was extracted from cell cultures grown under different light conditions (High Light (HL), Normal Light (NL), Low Light (LL), Dark (D) and iron starvation (−Fe)) and Northern blots hybridized with radiolabeled probes recognizing the small 25 nt product and the U6 snRNA as reference loading control. M, microRNA Marker (New England Biolabs, USA).

Among the *P. tricornutum* sRNAs localized in intergenic regions, the most abundant population of reads (>100,000 reads) is located on chromosome 2 (chr2: 29.039-29.123) (Additional file 2: Table S2). These sRNAs align to a 85 nt-long region that displays a very clear distribution of two overlapping 24 and 25 nt-long fragments, with a plateau at the center of the region (Figure 2A). This specific region concentrates around 8% of all the reads and 55% of the sRNA population matching intergenic regions. These abundant sRNAs have been detected in our five experimental libraries and in the independent dataset by Huang [28]. We can therefore exclude artifactual biases generated during library preparations or sequencing. Furthermore, their expression *in vivo* was validated by low molecular weight Northern-blot analysis using a 24 nt-long oligonucleotide complementary probe. In agreement with the sequencing data, the analysis detected 22-25 nt-long sRNAs from cells grown under different light conditions and iron starvation that appear as a smear in our analysis. A higher molecular weight band was also detected (Figure 2B) and presumably corresponds to the sRNA precursor. Therefore, like for the miRNAs, we made the hypothesis that these sRNAs could be the result of transcription and precise processing of a longer precursor. However, we failed to amplify this long transcript and to validate its sequence by 5′ and 3′-RACE PCR. Moreover, we did not succeed to predict any stable RNA secondary structure associated to a putative precursor sequence that might be processed to generate the smaller product. Therefore, the biogenesis of the most abundant *P. tricornutum* sRNAs remains to be determined. Interestingly, a conserved 25 nt-long fragment could also be recovered when analyzing the transcriptome of *T. pseudonana*[27] (Additional file 6: Figure S3A). As for *P. tricornutum*, this fragment aligns to an intergenic region of *T. pseudonana* genome on chromosome 7, suggesting the conservation of this novel small RNA population in diatoms.

### Novel populations of U2 snRNA-derived sRNAs

**Figure 3:**
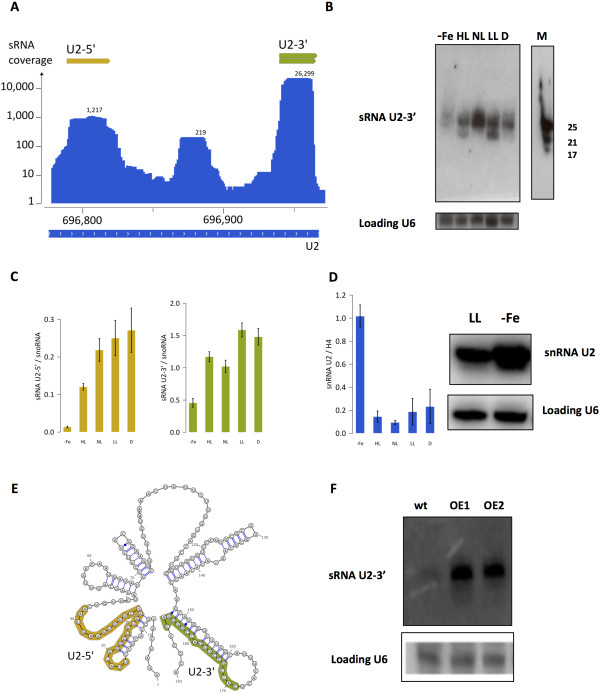
**A novel population of sRNAs derived from the U2 snRNA. (A)**. Screenshot of the reads profile associated to the U2 snRNA in chromosome 5 (chr5: 696.774–696.999). The U2-5′ (in orange) and U2-3′ (in green) sRNA populations associated to this region are indicated. **(B)** Validation of the U2-3′ expression by low molecular weight Northern blotting. Total RNA was extracted from cell cultures grown under different light conditions (High Light (HL), Normal Light (NL), Low Light (LL), Dark (D) and iron starvation (−Fe)) and Northern blot was probed using an oligonucleotide recognizing the U2-3′ sRNAs and the U6 snRNA as reference loading control. M, microRNA Marker (New England Biolabs, USA). **(C)** Relative transcript levels of U2-3′ and U2-5′ sRNAs determined by stem-loop qPCR, in cells grown under the same growth conditions described above. The expression values U2-3′ and U2-5′ were normalized to the snoRNA. Error bars are relative to three independent experiments. **(D)** Relative transcript levels of the U2 snRNA by qRT-PCR in cells grown under iron starvation and different light conditions. Normalization was done relative to histone H4 mRNA, used as a reference gene. Error bars are relative to three independent experiments. The U2 snRNA expression was also analyzed by Northern blot by using an oligonucleotide recognizing a region of the U2 snRNA upstream the U2-5′ small ncRNA, as a probe. **(E)** U2 snRNA secondary structure as annotated in Rfam [82] and the distribution of U2-5′ and U2-3′ highlighted on it. **(F)** Analysis of the U2-3′small RNAs in wild-type and overexpressing snRNA U2 lines (OE1 and OE2) by Northern blotting from cells grown under normal light condition. A total of 20 μg of RNA was loaded per lane and sRNA products were detected as described above.

Another sRNA population abundantly represented in our five libraries displayed a very clear distribution over a single 226 nt-long region (chr5: 696.774–696.999) that corresponds to the U2 snRNA gene (Figure 3). U2s are core components of the spliceosomes and are required for accurate pre-mRNA splicing in eukaryotes [42]. We found sRNAs along all the sequence, with two peaks corresponding to two different sRNA populations at the 3′ and 5′ ends of the region: one with a coverage of more than 26,000 reads, localized in the 3′ region of the U2 snRNA (named U2-3′), and another, with a much lower coverage of about 1,200 reads, at the 5′ end (named U2-5′) (Figure 3A). In order to validate their expression, we performed Northern blot analyses. An oligonucleotide recognizing the U2-3′ allowed confirming the existence of two sRNA populations of different sizes (23 and 25 nt), which were clearly detectable in cells grown in High Light (HL) and Low Light (LL). In the other tested conditions, Normal Light (NL) and Dark (D), only the upper 25 nt band was observed. Interestingly, in the iron starvation sample (−Fe) a clear reduction of both bands was observed compared to the other tested conditions (Figure 3B). Finally, the differential expression of the sRNAs matching the 3′ region of the U2 snRNA was further confirmed by stem-loop qPCR [43] (Figure 3C).

The U2-5′ sRNAs were not detected by Northern blot analysis, probably because of their low abundance compared to U2-3′ (Figure 3A) but their existence and their expression status were confirmed by stem-loop qPCR. The analysis revealed a reduced content of the U2-5′ expression under iron starvation, similar to what was observed for U2-3′ (Figure 3C). Interestingly, qRT-PCR and Northern blot analyses showed a reciprocal upregulation of the U2 snRNA in cells grown under iron starvation condition (Figure 3D). Taken together, these results strongly suggest that the U2-5′ and U2-3′ have a common biogenesis and their content is strongly reduced under iron condition, potentially through the inhibition of the processing of a U2 snRNA common precursor. Neither differences in the U6 snRNA expression nor generation of smaller RNAs associated to this other spliceosome core component were detected, strongly supporting the specificity of the processing generating U2-associated sRNAs. We then mapped the U2-5′ and U2-3′ sRNAs on the U2 snRNA secondary structure (Figure 3E). The U2-5′ is predicted to be part of stem-bulge structure, whereas the U2-3′ matches a hairpin-shaped stem loop-like secondary structure. This latter type of structure might be more efficiently processed to generate the highly expressed U2-3′ products. In order to test this hypothesis, we generated *P. tricornutum* transgenic lines over-expressing the U2 snRNA (see Methods). An increase in the U2-3′ steady-state level was observed in independent transgenic lines grown under normal light condition, providing a direct link between U2 gene expression and U2-3′ accumulation (Figure 3F). Remarkably, highly expressed small RNAs are also associated to the 3′ end of the U2 snRNA in *T. pseudonana* (Additional file 6: Figure S3B). This further supports the possibility that U2-3′ sRNAs are generated through the processing of the U2 snRNA precursor transcript through an unknown mechanism possibly conserved in different diatom species.

### Small RNAs associated to mature tRNAs

**Figure 4:**
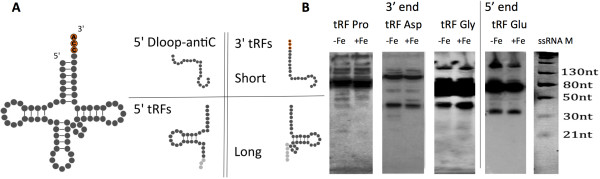
**sRNA associated to mature tRNAs. (A)** tRNA structure and the four types of sRNA fragments associated to tRNAs (tRFs) as observed from the sequencing of the different libraries. Variations in fragment lengths are represented in light gray. **(B)** Expression validation of selected tRFs by Northern blotting. Total RNA was extracted from cell cultures grown under iron starvation (−Fe) and normal condition (+Fe) in Low Light. Northern blots were hybridized using oligonucleotides recognizing the small tRF products. M, microRNA Marker (New England Biolabs, USA).

A novel and abundant class of regulatory sRNAs derived from the precise processing of mature tRNAs has been described in organisms as different as human [12, 38], mouse [44], protozoa [37, 45] and plant [46]. The sequencing of the *P. tricornutum* small RNA libraries performed in this study revealed a variety of sRNAs related to tRNAs in this microalga as well (Figures 1 and 4; Additional file 7: Table S3). In particular, different sRNA classes of different sizes were detected, likely resulting from different tRNA processing mechanisms: 1. Short fragments of around 19 nt, representing the majority of the *P. tricornutum* tRFs and found in all the libraries associated to the tRNA 3′ ends (3′-tRFs, 24 cases); 2. short tRFs of around 19 nt matching the tRNA D loop and the anticodon sequences, that do not fit in the general classification scheme of small RNA tRFs [47] (D loop/anticodon, 9 cases); 3. short tRFs associated to both regions, tRNA 3′ end and D loop/anticodon; 4. longer tRFs (30–35 nt), that were mostly detected under iron starvation (3′-tRFs: 12 cases, to 5′-tRFs: 6 cases) (Additional file 7: Table S3). A fragment matching the full length Glu^GAG^ tRNA from the 5′ end to the anticodon loop was also observed, and likely represents a putative half tRNA [48]. Interestingly, the 19 nt-long 3′-tRFs possess the CCA sequence that is added to the 3′ end of the trimmed tRNA intermediates in most eukaryotic organisms [12]. On the contrary, the longer 3′-tRFs do not present the CCA sequence, hinting at the fact that the two 3′ tRFs may originate from different RNA processing mechanisms. tRNA-associated small RNAs have also been detected with a similar proportion (2-4%) in the *T. pseudonana* transcriptome and with a particular enrichment in 19 and 25 nt-long fragments (Additional file 6: Figure S3C).

We experimentally validated the expression of a total of four *P. tricornutum* 3′- and 5′-tRFs that showed the highest coverage (Figure 4 and Additional file 7: Table S3) using probes that cover both the short and the long fragments. For all the tested tRNA fragments, we detected by Northern blot analysis the tRFs of 30–35 nt and longer 80–100 nt fragments that may likely represent the mature tRNAs. For the tRF-Asp^CAG^ and tRF-Pro^CCT^, a slightly increased expression in cells grown under iron starvation was also observed (Figure 4), which is consistent with the transcriptome analyses. Contrariwise, we had difficulties to detect the shorter tRFs for the tested tRNAs, that become visible only after long exposure of the membranes.

### Small RNA-directed control of transposable elements expression

**Figure 5:**
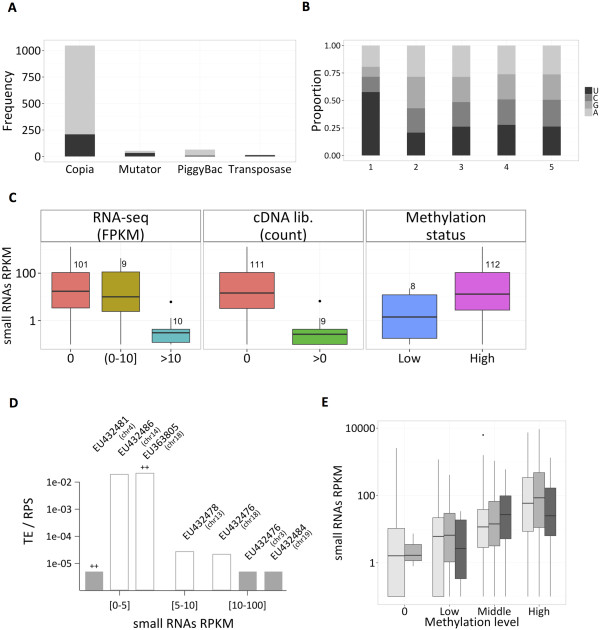
**Properties of sRNAs covering transposons. (A)** Repartition of the sRNA sequences in the different diatom transposon families (only for TE annotation above 300 bp). We report the number of transposons with a sRNA coverage above 5 RPKM (grey) or with a low or no sRNA coverage (black). **(B)** 5′ end nucleotide composition of the reads (from position 1 to 5) aligning to transposons. **(C)** Relationship between sRNA coverage and autonomous TEs expression or autonomous TEs methylation. From left to right: transcript expression according to RNA-seq evidence, or cDNA libraries, and methylation status in transposons. **(D)** Experimental validation of the transcriptional activity level of autonomous transposons by qPCR. The transposons are grouped on the x-axis with an increasing coverage in sRNAs: low (sRNA RPKM < 5), middle (5 < RPKM < 10) and high (RPKM > 10) coverage. A grey shaded box is reported when the product could not be amplified. Autonomous transposons whose expression is supported by both EST and RNA-seq evidence are annotated with the symbols “++”. Labels above bins correspond to the Genbank identifier and localization of the transposons. **(E)** Comparison of methylation level and sRNA coverage on all Copia-type LTR transposons longer than 50 nt. Boxes are colored according to the length of the transposons region: 50 to 1000 nt (light grey), 1000 to 4000 nt (grey) and more than 4000 nt (dark grey). Methylation level is computed as the proportion of methylated probes that overlap the transposon region.

The majority of small RNA fragments correspond to repetitive elements (Table 1) and 98% of the reads that align to repeat regions cover diatom LTR retrotransposons (LTR-RTs) [25], the most abundant TE family in diatom genomes. These fragments tend to have a length between 25 and 30 nt, cover the entire TE sequence, and are equally distributed along both strands. We observed a consistent coverage of the TE regions across all five libraries (Spearman correlation > 0.89 between any 2 libraries). The sRNA coverage was thus summarized on a region using RPKM [49], a metric normalization for region lengths and sequencing depth. Most of the regions covered above background level were Copia-like or PiggyBac like TEs (Figure 5A). Nucleotide composition of the reads appears biased at their 5′end, with U being the first nucleotide on almost 60% of the reads (Figure 5B).

Various classes of siRNAs and piRNAs have been reported to regulate the expression of TEs in eukaryotes, either post-transcriptionally or through cytosine DNA methylation and/or other chromatin modifications [50]. To assess if the population of *P. tricornutum* sRNAs covering the TEs may also have a regulatory role, sRNA coverage was compared to the expression level of the autonomous TEs [25]. Information regarding TE expression was obtained from two independent sources, a set of EST libraries [51] and a recently published RNA-Seq dataset [26] both obtained from cells grown under normal light conditions. Therefore, to facilitate further comparisons, we only considered in the following analyses our sRNAs dataset obtained from cells grown under Normal Light condition. For both RNA-seq and EST datasets, the small RNA coverage is significantly higher on TEs with undetectable or low expression (Mann-Withney one sided test, RNA-seq: pvalue < 4e-5, EST: pvalue < 5e-3) (Figure 5C). This suggests that sRNA abundance correlates with TEs characterized by low or no expression, possibly triggered by efficient repression or silencing. This hypothesis was tested by RT-qPCR using primers specific to 7 selected transposons with high (3), medium (1), or low (3) sRNA coverage, respectively. This revealed that autonomous TEs with medium or high sRNA coverage have an expression level at least 1,000 folds lower than the ones with low or no coverage (Figure 5D). This inverse correlation further supports the hypothesis that sRNAs may contribute to the silencing of these TEs. Furthermore, two out of the three TE transcripts with high sRNA coverage could not be amplified, and may therefore be fully silenced.

### The distribution of sRNAs associates with DNA methylation in *P. tricornutum*

Recently, a reference methylome has been established in *P. tricornutum* by genome-wide profiling of 5-methyl-cytosine [26]. The analysis revealed extensive DNA methylation over transposable elements, which correlated with low expression levels [26]. We made use of this resource to untangle a possible link between the presence of sRNAs and DNA methylation over transposable elements (TEs), as previously described in other organisms [52]. A total of 359 sRNA producing regions overlap methylated TEs, altogether corresponding to 6% genome coverage. These regions are on average longer than 4,000 bp and are covered by 56% of the uniquely mapping reads (Additional file 1 Table S1). Correlation analyses further show that high DNA methylation levels associate with elevated sRNA coverage on autonomous TEs (Mann-Withney one sided test, pvalue < 8e-3 in Figure 5C; Spearman rank correlation = 0.41 in Figure 5E), suggesting that sRNAs could direct DNA methylation at TEs as previously reported in other organisms [52] (Figure 5E). To further test this hypothesis at the genome-wide level, we further searched for a possible link between TE-associated sRNAs and DNA methylation of all annotated Copia-type transposons on assembled chromosomes (1544 TEs of length > 50). This revealed that the large majority (74%) of the methylated transposons has an RPKM coverage of sRNAs higher than 10 (Figure 5E). The effect is significantly more pronounced on long TEs, with 95% of methylated TEs longer than 1,000 nt (338 TEs) having a RPKM higher than 10. Taken together, these data are fully supportive of a model in which sRNAs direct DNA methylation at many TE loci in *P. tricornutum*.

Methylated DNA is mainly found on transposable elements in the *P. tricornutum* genome [26]. However, high DNA methylation levels could also be observed on some coding genes, possibly associated with their transcriptional repression [26]. By comparing the methylation signals encompassing genes with the sRNAs coverage, we also observed a significant positive correlation between methylation levels and sRNAs abundance (Pearson correlation = 0.24) (Additional file 8: Figure S4 and Table 1). This observation further supports a possible role of sRNAs in determining the methylation state of a DNA region on both TE and gene loci. Two main points can be remarked: 1. about 83% of the 3380 Highly Methylated Regions (HMR) overlap with a sRNA-producing locus (76% overlap on more than 75% of their length) and, 2. there is a striking periodic pattern of read placement on the HMRs (Figure 1, right bottom). Fast Fourier Transform analysis was realized on HMRs and a significant period of 180 nt (at times of 90 nt) was revealed (Additional file 9: Figure S5 and Additional file 1: Table S1). Conversely, we did an unconstrained search for regions where the read coverage would have at least three significant periods within a 5000 bp window. We detected 270 regions, which are mostly overlapping HMR (86% or 233 regions) and with an average periodicity between 175 and 225 nt (72% or 196 regions). In the analysis performed, this periodic signal was significantly more pronounced on the HMRs associated with coding genes than on TE regions (Additional file 10: Figure S6A). This first observation rules out the possibility that we observed an artifact generated by repeated regions. As a matter of fact, TE repeats are located in different genomic positions. There, ambiguous mapping of sRNA reads, not necessarily lying on phase, would blur the periodic patterns in coverage signal (see for example Additional file 10: Figure S6B). In conclusion, these analyses permitted to uncover an unprecedented periodic organization of sRNA distribution over multiple genes and TEs heavily targeted for DNA methylation.

## Discussion

Although the annotation of sRNAs in almost all studied organisms is becoming more and more comprehensive, these regulatory elements have only recently been identified in unicellular and multicellular algae belonging to different lineages [53–58]. In this work, we have significantly extended the characterization of the sRNAs from a model Stramenopile species, the marine diatom *P. tricornutum*, providing new information regarding diversification and function of these elements in eukaryotes. The genome-wide analysis of sRNAs, obtained from cells grown under different light and nutrient stress conditions, revealed the existence of a variety of sRNA populations with characteristic lengths and specific distribution patterns on diverse genomic locations (Figure 1). The functional and *in silico* characterization of these elements allowed us to reach several novel conclusions.

We questioned the presence of canonical miRNAs in diatoms, previously reported in *P. tricornutum*[28]. We used a computational approach to look for putative miRNAs in our small RNA dataset, but none of our attempts revealed canonical pre-miRNA-like hairpins that could be substrates for a Dicer-like enzyme. Moreover, none of the sRNAs we identified share significant sequence similarity when compared with microRNA sequences from miRBAse [41] and miRNAs from *Ectocarpus siliculosus*, a Stramenopile multicellular brown alga that represents the only example reported for this lineage in the literature with experimentally validated microRNAs [55, 56]. These results strongly indicate that canonical miRNAs, such as those characterized in different eukaryotic organisms so far, are not major regulators of gene expression in *P. tricornutum*. However, at this stage, we do not rule out the possibility that canonical miRNAs could be identified in response to other environmental cues and/or through a more important small RNA sequencing coverage than the one used in the present study. Nevertheless, we were able to identify some sRNAs located on stable precursor secondary structures, which we designated as miRNA-like because they possess only a part of canonical miRNA features. In particular, an interesting polycistronic miRNA-like cluster has been identified on chromosome 10, with a RNA stem containing a predicted miRNA locus, consistently detected in all our libraries, and another miRNA-like, on another RNA stem, that is observed only in cells under iron starvation conditions. The data suggests that under different environmental conditions, primary transcripts might be differentially processed to generate different miRNA-like elements with different function. Similar examples have been already reported in human [29]. Interestingly, even if these miRNA-like are well represented and predicted to originate from stable structures, we identified 2 nucleotide 3′ overhangs at only one strand of the putative dsRNAs. This observation suggests that this type of small RNAs might be generated via a Dicer-independent mechanism [59, 60]. Alternatively, the *P. tricornutum* Dicer-like enzyme, which shows a partial sequence conservation with plant and animal Dicer homologues and possesses only a dsRBD domain followed by 2 RNAs III domains [30] might bind and cleave the opposite strands of a dsRNA in an atypical way to generate the miRNA-like molecules identified in this study. Therefore, further characterization of these miRNA-like elements will likely help identifying novel sRNA biogenesis pathways in diatoms.

Our dataset allowed us to identify the most abundant population of sRNAs in *P. tricornutum*, showing a clear distribution peak of 24-25 nt in length, localized on an intergenic region of chromosome 2 (chr2: 29.039-29.123). Northern blot analyses experimentally demonstrated the existence of sRNAs in *P. tricornutum* for the first time (Figure 2B). Their detection in cells grown under different conditions excludes a non-specific RNA degradation origin. Similarly to the miRNAs, these sRNAs may result from the processing of longer RNA precursors, which could successfully be detected. However, despite numerous attempts, we did not succeed to amplify and validate the primary transcript and to identify the dsRNA structure that may generate these products. We can thus hypothesize that, differently from the miRNAs, the precursor is not transcribed by the RNA polymerase II [61] or is subjected to other 5′ or 3′ end modifications (e.g., decapping and subsequent 5′-3′ degradation), that preclude their amplification using standard RACE. Interestingly, these sRNAs share sequence similarities with sRNAs matching an intergenic region of the *T. pseudonana* genome. This suggests that novel sRNA populations of unknown origin are conserved between different diatom species and likely evolved to play similar regulatory functions in these microalgae.

Novel results derived by next-generation sequencing experiments have provided some indications about the existence of sRNAs associated to spliceosomal snRNAs [62] and in mRNA length regulation (telescripting) [63]. The results presented in this study unveiled a previously unknown regulatory network involving U2 snRNA-derived sRNAs. Two different sRNA populations at the 5′ and 3′ ends of the U2 snRNA (the U2-5′ and U2-3′ sRNAs) were identified. We validated the expression of both U2-5′ and U2-3′ and provided functional evidences that the full length U2 RNA represents the primary precursor of the sRNA populations. Namely, the reported increase in U2-3′ content in transgenic lines over-expressing U2 snRNA robustly supports this possibility. Of particular interest, the expression of both U2-5′ and U2-3′ is strongly repressed in cells grown under iron starvation condition. Interestingly, an induction in the U2 snRNA transcript was observed in the same condition. This negative correlation supports the existence of a regulatory network balancing the reciprocal expression of the U2 snRNA and the U2-derived sRNAs. An increase in the U2 snRNA might be needed to control the cellular responses to deficiency of iron, a critical nutrient for life in the ocean [32, 33, 64, 65]. Likewise, we can also hypothesize a regulatory role played by the U2-derived sRNAs in controlling *P. tricornutum*’s response to iron availability. Finally, a feedback loop controlling the expression of U2 snRNA precursors by the associated sRNAs might also occur. Interestingly, highly expressed U2-3′ smallRNAs have been detected also in *T. pseudonana,* strongly supporting a conserved biogenesis of these smallRNA populations in diatoms*.* The characterization of this complex regulatory process, the identification of U2-3′ mode of action possibly involving sequence-specific targeting, the putative interaction between these sRNAs and the splicing machinery, all represent important questions to address in the future.

Similarly to what was reported in the last years in many organisms [38, 66], we identified a variety of sRNAs associated to tRNAs also in *P. tricornutum* and *T. pseudonana* (Additional file 6: Figure S3C and Additional file 7: Table S3). These sRNAs possibly derive from different processing mechanisms and endorse different cellular functions. In *P. tricornutum,* we found putative tRFs of around 19 nt, mostly corresponding to the tRNA 3′ ends and carrying the trinucleotide CCA at the acceptor stem, which likely derive from tRNA cleavage in or at the T loop [48]. We also detected short fragments associated to the D loop and to the anticodon of 9 different *P. tricornutum* tRNAs (Additional file 7: Table S3). Because they do not fit into the general classification scheme [48], we are not able to anticipate the processing events generating these short tRFs from precursor or mature tRNAs. Moreover, we detected several longer small RNAs (30-35 nt) that correspond to both 5′ and 3′ parts of full length tRNAs, and probably derive from cleavages at different positions (Figure 4). Some of these long fragments might be classified as half tRNAs (e.g., Glu^GAG^ tRNA). In other species, such tRNA fragments, usually referred to as ‘tRNA, stress-induced small RNAs’, derive from the tRNA cleavage in the anticodon loop, and have been shown to regulate a wide range of cellular processes (e.g., translational efficiency and mitochondrial-mediated apoptosis [48, 66–69] in response to nutritional, biological, or physicochemical stresses). Interestingly, our data also unveil an enrichment of the long tRNA fragments in *P. tricornutum* cells grown under iron starvation (Additional file 7: Table S3). The data suggest that cells exposed to iron deficiency may differently modulate the processing of specific tRNAs, thereby generating fragments of different sizes that may play different roles in the control of diatom responses to nutrient limitation. We validated the expression of selected sRNAs associated to tRNAs by Northern blot and in agreement with the sequencing data, we observed different populations of tRNA fragments (Figure 4). In general, the detection of the shorter tRFs appeared more problematic in Northern blot analyses as compared to the longer tRFs and to mature tRNAs, likely due to the different stability of these products.

In this study, we also dedicated particular attention to the characterization of sRNAs matching TEs and repeats, which represent almost half of the *P. tricornutum* small RNA repertoire. TEs are considered an important driving force for genome evolution by inserting at various genomic locations and altering gene expression and regulatory functions [70, 71]. A significant part of diatom genomes is devoted to TEs and repeat sequences, particularly in *P. tricornutum*[23]. Moreover, a comparative genomic analysis between *P. tricornutum* and the centric diatom *T. pseudonana* revealed that TEs have been important contributors to the genome divergence between the two diatom species, with *P. tricornutum* having undergone a significant amplification of TE sequences as compared to *T. pseudonana*, respectively 6.4% and 1.9% of the genomes [23, 25]. Interestingly, 7 to 15% of the sRNAs are matching repetitive sequences in *T. pseudonana*[27], a proportion significantly lower than in *P. tricornutum*. These observations suggest that the increased amplification of TEs in *P. tricornutum* is likely responsible for the over-representation of endogenous sRNAs associated to TEs in this species. Such a mechanism would be consistent with recent findings in Arabidopsis whereby gradual enhanced accumulation of siRNAs derived from the retrotransposon Evadé were also observed upon progressive amplification of this TE in the genome [72]. TE-derived sRNA species likely regulate the expression/transposition of these mutagenic sequences in normal growth conditions but may also control the transposition events under selective conditions thereby contributing to genome evolution and adaptation. Consistently with this concept, expression of some LTR retrotransposons (LTR-RTs) can be modulated by different stresses in *P. tricornutum*, suggesting that the mobilization of some TEs may contribute to genome rearrangements [25]. The likely essential role of TEs in genome evolution and adaptation, and the high representation of TE-derived sRNAs in *P. tricornutum* therefore prompted us to explore the possible regulatory role of these sRNA species. Similarly to other organisms, our data clearly indicated that a high sRNA coverage correlates with low TE expression (Figure 5), suggesting that the sRNAs indeed direct TE silencing. We gained more insights into the mechanisms by which sRNAs could direct TE silencing by comparing our small RNA profiling with the methylated regions obtained from a recent genome-wide analysis generated in the same species [26]. This actually confirmed that most of the TEs targeted by sRNAs are characterized by high methylation levels, further supporting the possibility that these ncRNAs contribute to the establishment of DNA methylation at these TEs and to their transcriptional or post-transcriptional silencing. We then extended the analysis of small RNA-associated DNA methylation to coding regions. Interestingly, as already observed by Veluchamy *et al.*[26], a high methylation level was observed in genes that are specifically covered by sRNAs (Figure 1, Additional file 1: Table S1 and Additional file 8: Figure S4). Our data further show that these sRNAs have sizes and distribution patterns like TE-associated sRNAs, suggesting that they are also generated via similar mechanisms. Interestingly, we already showed in a previous study [30] that *de novo* cytosine methylation is associated with transgene silencing induced by artificially expressing antisense and inverted-repeat RNAs in *P. tricornutum.* Altogether, this information suggests that a small RNA-dependent DNA methylation process is widely used by diatoms not only to control the expression of TEs but also of transgenes as well as protein-coding genes by presumably directing DNA methylation within these genetic elements. These results also open interesting novel questions on the evolution, distribution and function of such epigenetic-based regulatory pathways in different organisms. In mammals, DNA methylation occurs throughout the genome, and many genes are body methylated, although this methylation pattern has not been associated with the presence of sRNAs [73]. In wild-type Arabidopsis plants, gene body methylation has also been reported for many genes, but this methylation occurs in a small RNA-independent manner and appears to be irrelevant for silencing [74]. By contrast, loss-of-function mutations in some active Arabidopsis DNA demethylase mutants were reported to enhance DNA methylation in specific gene body regions through siRNA-mediated DNA methylation, presumably leading to their silencing [75, 76] and supporting a functional role for siRNA-directed gene body methylation in these mutant backgrounds. Therefore, we can hypothesize that different photosynthetic organisms belonging to different lineages had the potential to use the small RNA-dependent DNA methylation pathway to control expression of coding genes, although in plants this effect is solely appreciated in specific mutants but not in wild-type plants grown under standard conditions. Furthermore, the sRNA distribution and DNA methylation profile over gene bodies differs significantly between diatoms and plants (Figure 1 and Additional file 8: Figure S4) [75]. Therefore, the observations made on *P. tricornutum* will provide fertile ground for new exciting insights into the mechanisms involved in small RNA-directed gene body methylation and into the eventual effects of such epigenetic modification on transcription initiation and elongation.

In addition to the likely functional relevance of *P. tricornutum* TE-associated sRNAs in DNA methylation and transcriptional gene silencing, we found that these sRNA species were 25 to 30 nt in length and have a 5′ uridine bias (Figure 5). Interestingly, these features are shared with animal piRNAs suggesting a potential link between these silencing pathways. However, our analysis did not reveal self-complementary sequences, characteristic of a Ping-Pong amplification, and no canonical PIWI protein could be retrieved in the *P. tricornutum* genome (data not shown), arguing against the presence of a canonical piRNA biosynthetic pathway in this marine microalga. Future in depth characterization of such diatom small RNA species will thus be necessary to compare and contrast these sRNAs with animal piRNAs.

The periodic distribution of sRNAs over methylated regions represents a completely novel hint. This phenomenon is characterized by a 180 nt-long period (at times of 90 nt) that is sharp on coding genes and also visible with high confidence on TEs. At this stage, it is still difficult to predict the mechanisms generating such a peculiar distribution pattern or to link sRNA distribution with DNA methylation activities and transcriptional silencing. In future studies, it will be relevant to assess whether periodic sRNA distribution correlates with local chromatin properties such as nucleosome occupancy, histone post-translational modifications or histone variants, which have not been mapped in diatoms so far. More specifically, it might be interesting to test whether the 180 nt periodic enrichment of sRNAs matches inter-nucleosomal domains with about the same size, possibly reflecting the capacity of RNA Polymerases producing small RNAs to efficiently transcribe these more accessible regions in a condensed and silenced chromatin context. Reciprocally, on protein-coding genes, sRNAs might also target inter-nucleosomal domains more easily than histone-wrapped nucleosomal DNA, particularly in a non-replicative context. Interestingly, the existence of periodic cytosine methylation has recently been detected in microalgae with compact nuclei and strong gene body methylation, regardless of gene expression levels. In *Ostreococcus lucimarinus* and *Micromonas pusilla*, the periodic DNA methylation occurs specifically over nucleosome linker (inter-nucleosomal) regions, and appears to contribute to genome architecture and nucleosome positioning [77]. Based on our observations, it might be interesting to test whether sRNAs also display a periodic enrichment at the same loci in these species. Of note, regularly spaced methylation has not been detected in *P. tricornutum*[77]. This might reflect the different methylome features between *P. tricornutum* and these other algae*.* Alternatively, more refined DNA methylation mapping might be required to determine whether periodic DNA methylation and sRNA distribution overlap at some TEs or gene loci in *P. tricornutum*.

The analysis of diatom genomes reveals the presence of genes encoding several proteins that might be required for generating DNA methylation-associated sRNAs. *P. tricornutum* and *T. pseudonana* genomes both encode Dicer- and Argonaute-like proteins and several putative RNA-Dependent RNA Polymerases (RdRPs) but no PIWI protein [30]. These proteins might be responsible for generating sRNAs, for amplification mechanisms and for the possible recruitment of DNA methyltransferases (DNMTs) identified in previous studies [25, 77]. Considering the peculiar genome of diatoms, their evolutionary history, and the information on the sRNAs landscape from this study, we also expect that additional, still unknown, components participate in the small RNA-based regulation of diatom genome function. Their characterization will likely provide novel insights into the genetic bases allowing the successful proliferation of diatoms in the oceans and will help addressing relevant questions about the conservation of these processes in organisms that cover different branches of eukaryotic evolution.

## Conclusions

This comprehensive characterization of the *P. tricornutum* small RNAs landscape uncovered an unexpectedly complex composition of the sRNA complement in diatoms. While miRNAs seem not to play a major regulatory role in *P. tricornutum*, other ncRNA types with well-described function, such as tRNAs and U2 snRNA, represent a major source of sRNAs and could play important functional roles in *P. tricornutum*’s responses to environmental stresses such as iron starvation. This discovery also implies that complex and interconnected networks of ncRNA pathways exist in diatom cells, and their fine-tuning is likely to be fundamental for the biology of these organisms and their extraordinary ability to proliferate worldwide. The existence of highly expressed sRNAs of unknown origin and conserved in two distantly related diatom species also indicates that diverse small RNAs sources await to be discovered in these algae. The most represented population of sRNAs match TEs and likely contributes to their transcriptional silencing through a yet-to-be characterized RNA-dependent DNA methylation mechanism. Protein-coding genes might also be subjected to similar regulatory processes for down regulating their expression at a transcriptional, and possibly also at a post-transcriptional level. In conclusion, this extensive characterization of *P. tricornutum* small ncRNAs can contribute to future studies aiming at understanding diatom biology, the molecular bases underlying their ecological success in the contemporary oceans, and their capacity to optimally perform in highly changing environments. The periodic pattern of sRNAs distribution associated with genic DNA methylation unveiled here, represents an unexpected discovery that opens the way to search for similar periodic features in other eukaryotic groups.

## Methods

### Cell cultures conditions

The CCMP632 strain of *P. tricornutum* Bohlin was obtained from the Provasoli-Guillard National Center for Culture of Marine Phytoplankton. Cultures were grown in f/2 medium [78] at 18°C under white fluorescent lights in 12 h light: 12 dark cycles. For the preparation of the different small RNA libraries, cells were grown under different conditions: Low Light (LL) (30 μmol photons m^−2^ s^−1^), Normal Light (NL) (80 μmol photons m^−2^ s^−1^); in dark for 60 hours (D) and then shifted to High Light (500 μmol photons m^−2^ s^−1^) for 5 hours (HL) and under iron starvation (iron 5nM). For the iron starvation, cultures of *P. tricornutum* were grown at low light, in Artificial Sea Water medium [64], containing 5 nmol · liters^−1^ Fe (Fe-limited) as final concentration. In all the experiments, cells in the exponential phase of growth have been used.

### Small RNA library preparation and sequencing

In all conditions (NL, HL, LL, D and iron starvation), total RNA was extracted from diatom cells using the Trizol method (Invitrogen, USA). sRNAs were separated by size fractionation on denaturing polyacrylamide gels and then fragments in the 18–35 nt size range were purified. The RNA samples were sent for cDNA synthesis and Illumina sequencing to Vertis Biotechnologie AG company (http://www.vertis-biotech.com). The purified RNAs were poly(A)-tailed and ligated using a RNA adapter to the 5′-phosphate of the small RNAs. First-strand cDNA synthesis was performed using an oligo(dT)-adapter primer and M-MLV RT (Ambion), then followed by PCR amplification. The resulting cDNA populations were sequenced on an Illumina sequencer. The raw sequence data can be accessed at the NCBI Sequence Read Archive under the accession number SRX648639.

### Read mapping and primary data analyses

The reads coming from the 5 libraries associated to different environmental conditions were aligned to the *P. tricornutum* genome with MicroRazerS [79] and subsequently quality filtered (Additional file 5: Supplementary Methods). Read coverage was computed in two ways, either by considering only unique matches, or by reweighting each read according to the number of matching locations. Unless stated otherwise, we considered unique matches in our analyses. All data analyses were done using a combination of python, R and shell scripts, and genome arithmetic tools [80, 81].

Using as genome reference Phatr v2.0 from JGI [23], gene annotation was derived from EST expression data (libraries os, sp, sm and om referenced in [51]), and non-coding RNA annotation from RFam v11.0 [82]. Additionally, tRNA genes were all reannotated using tRNAscan [83]. RNA-Seq expression levels and methylation data were obtained from [26]. Due to the large dynamic range gained by the RNA-Seq, we define a category of lowly expressed genes at the 15th percentile of all genes expression. Within a genomic interval, the methylation level is reported as the proportion of probes called as methylated on that interval.

The list of autonomous transposable elements was derived from [25] and realigned using LAST [84], resulting in the identification of 119 autonomous regions on the genome. The transcription level of an autonomous transposon (EST or RNA-Seq evidence) was deduced from the expression level of the gene models overlapping it, taking the highest value.

Prediction of RNA secondary structures was done with RNAfold [85] and visualization with Varna [86]. To account for the natural diatom environment, we also lowered the default folding temperature to 24°C.

### Prediction of processed elements

miRNA-like elements were predicted on a set of regions obtained by clustering reads that aligned less than 100 bp from each other, and covered by at least 10 reads (called sRNA producing loci).

Then loci, where the aligned reads profile is compatible with bona fide small ncRNA processing, were selected according to a set of published and empirical criteria [34, 35]. All the filtering steps are described in details in Additional file 5: Supplementary methods. In summary, we selected loci with at least one strand-specific, highly localized fragment in the 19-29 nt range in a fashion like what is classically used for prediction in plants and animals high-throughput sequencing data [87–89]. The set of identified loci is then merged across the five libraries by taking the union of all genomic intervals, resulting in 50 candidate loci.

When analyzing a previously published dataset of small RNA sequences [28], SRA: GSM72456[0–2], we used the same strategy for sequence alignment and annotation of the candidate regions. The small RNA sequences coming from an experiment on *T. pseudonana*[27] (SRA027146, SRA027168) were also aligned with the same strategy on the reference genome. We predicted miRNAs with MIReNA v2.0 [29], recently shown to perform amongst the best methods for *de novo* miRNA annotation [36]. We used our list of candidate regions as putative precursors –excluding known non-coding RNAs. The 20 putative precursors are obtained by extending the candidate region by 50 nt on both sides, and the putative mature fragments are proposed amongst the 6 more abundant in the region. We additionally evaluated the significance of the MFE *m* associated with the predicted fold by counting the proportion of samples with an MFE lower than *m* over 1000 random regions of the same size drawn from *P. tricornutum* genome. The derived empirical pvalue is then deemed significant when below 5%.

### Analysis of the periodic distribution of reads along the *P. tricornutum*genome

To detect and quantify periodic distribution of read coverage, we performed analysis based on Fast Fourier Transform. Regions with periodic distribution are detected by sliding a window of length 1000 bp (100 bp increments) along each chromosome. In each window, a Fast Fourier Transform (FFT) is computed based on the profile of read coverage and including multimapping reads. The main period is extracted together with the corresponding FFT coefficient, normalized by the total signal. Windows with a coefficient higher than 75% of the maximal value are annotated with a significant period.

### Northern blot analysis of low molecular weight RNAs

Total RNA was isolated as described above. Northern blot analyses of low molecular weight RNAs were performed by separating 20 μg of total RNA on 14% acrylamide gels. RNAs were transferred to N+ membranes (Amersham Bioscience) by using a semi-dry system (BioRad). For the detection of small RNAs, membranes were hybridized overnight with oligonucleotides complementary to the small RNA sequences. The probes were end labeled with γ-^32^P-ATP using T4 PNK (New England Biolabs, Beverly, MA) in PerfectHyb Plus hybridization buffer (Sigma). Different temperatures were used for the hybridization according to the different Tm of the probes. The membranes were washed twice for 20 min with 2× SSC, 1% SDS at 40°C, and then scanned on a Typhoon Imaging System 9200 (GE Healthcare) or exposed to x-ray films.

### Analysis of sRNAs by stem-loop qPCR

The expression profiling of selected sRNAs was also analyzed with the Stem-loop Real-time reverse transcriptase polymerase chain reaction (RT-PCR) [43]. In brief, 2 μg total RNA were reverse transcribed with specific RT-primers, and the resulting cDNA was subjected to real-time reverse transcription quantitative qRT-PCR with a SYBR Green I assay (Roche SYBR Green I Master), using specific sRNA forward primers and the universal reverse primer. The primers were designed according to published criteria [90, 91] and are specified in Additional file 11: Table S4. The small nucleolar RNA SnoR85 was used as endogenous reference. In our analyses, quantification of each small RNA relative to snoRNA was calculated using the following equation: N = 2^−ΔCt^, ΔCt = Ct_smallRNA_ − Ct_snoRNA_. All reactions were carried out in triplicate.

### Characterization of the full length snRNA U2 by 3′ and 5′ RACE-PCR

The sequence of the snRNA U2 full-length transcript was determined by FirstChoice® RLM-RACE Kit (Invitrogen™). cDNA was amplified in accordance with the manufacturer’s instructions. The primers used in the 5′ and 3′ RACE PCR are specified in Additional file 11: Table S4. RACE products were cloned into TOPO® PCR vector (Life Technologies) and sequenced.

### Expression analysis of snRNA U2 and TEs

RNA extraction and quantitative PCR (qRT-PCR) were performed as described in De Riso *et al*. [30]. The cDNA was quantified using a SYBR Green qPCR kit (Roche LightCycler 480 SYBR Green I Master) and gene specific primers. The RPS housekeeping gene (ribosomal protein small subunit 30S; Protein ID 10847) or the histone H4, were used as reference genes (Additional file 11: Table S4). All reactions were carried out in triplicate.

### Construction of vectors for U2 sRNA overexpression

The vector for the U2 snRNA overexpression was generated using standard molecular cloning procedures. The full length U2 snRNA was amplified by PCR from the Pt1 cDNA using the following primers *OE U2snRNAFw* (XbaI site in italic): ctag*TCTAGA*TTCGCCTTATTGGCTTTGAT and *OE U2snRNARv* (EcoRI site in italic): ccg*GAATTC*gagggaAAAGTGGGGGTACA. The fragment was cloned downstream a phleomycin resistance cassette (*Sh ble* gene), and under the strong FcpF promoter, as described in [92].

The vector was introduced into wild-type *P. tricornutum* strain by microparticle bombardment using a BiolisticPDS-1000/He Particle Delivery System (Bio-Rad) [93] and transgenic lines were selected on 1% agar plates (50% f/2 medium) containing 100 μg/mL Phleomycin (Invivogen) in Normal Light (80 μmol photons m^−2^ · s^−1^). The presence of the construct in the transgenic lines was verified by checking the presence of the *Shble* gene by PCR with the primers Shble1fw and Shble1 rv. The presence of the full length U2 snRNA was verified by performing PCR using the primers Shble1 fw and snRNAU2 rv (Additional file 11: Table S4). On the selected transgenic lines, Northern blot analysis was performed, as described before. For the detection of the sRNAs, the membrane was hybridized overnight with oligonucleotides complementary to the U2-3′ sequence.

### Availability of supporting data

The raw sequence datasets supporting the results of this article are available in the sequence read archive repository (http://www.ncbi.nlm.nih.gov/sra/) under the accession number SRX648639. Additional supporting methods, figures and tables are included as supplementary files.

## Electronic supplementary material

Additional file 1: Table S1: Summary of the properties of all potential sRNA producing loci. (PDF 42 KB)

Additional file 2: Table S2: List and properties of regions annotated with our set of filters for miRNAs identification. (PDF 44 KB)

Additional file 3: Figure S1: Predicted secondary structures and read profiles for a miRNA-like candidate on chr10 predicted by MIReNA. (PDF 295 KB)

Additional file 4: Figure S2: Predicted secondary structures and read profiles for a miRNA-like candidate on chr1 predicted by MIReNA. (PDF 292 KB)

Additional file 5: **Supplementary data and methods.** (DOCX 58 KB)

Additional file 6: Figure S3: Small RNA fragments expressed in *T. pseudonana.* (PDF 158 KB)

Additional file 7: Table S3: Properties of tRNA associated reads. (PDF 66 KB)

Additional file 8: Figure S4: sRNA coverage and methylation level on genes. (PDF 57 KB)

Additional file 9: Figure S5: Distribution of the main period in sRNA coverage on the set of HMR regions longer than 1000 bp (351 regions). (PDF 41 KB)

Additional file 10: Figure S6: Example of regions with periodic sRNA coverage detected. (PDF 1 MB)

Additional file 11: Table S4: List of primers utilized in the present work. (PDF 39 KB)

## References

[1] Ghildiyal M, Zamore PD (2009). Small silencing RNAs: an expanding universe. Nat Rev Genet.

[2] Gomes AQ, Nolasco S, Soares H (2013). Non-coding RNAs: multi-tasking molecules in the cell. Int J Mol Sci.

[3] Mattick JS (2001). Non-coding RNAs: the architects of eukaryotic complexity. EMBO Rep.

[4] Jacquier A (2009). The complex eukaryotic transcriptome: unexpected pervasive transcription and novel small RNAs. Nat Rev Genet.

[5] Rinn JL, Chang HY (2012). Genome regulation by long noncoding RNAs. Annu Rev Biochem.

[6] Grimson A, Srivastava M, Fahey B, Woodcroft BJ, Chiang HR, King N, Degnan BM, Rokhsar DS, Bartel DP (2008). Early origins and evolution of microRNAs and Piwi-interacting RNAs in animals. Nature.

[7] Kiss T (2002). Small nucleolar RNAs: an abundant group of noncoding RNAs with diverse cellular functions. Cell.

[8] Ambros V, Chen X (2007). The regulation of genes and genomes by small RNAs. Development.

[9] Eggleston AK (2009). RNA silencing. Nature.

[10] Molnar A, Melnyk C, Baulcombe DC (2011). Silencing signals in plants: a long journey for small RNAs. Genome Biol.

[11] Carthew RW, Sontheimer EJ (2009). Origins and Mechanisms of miRNAs and siRNAs. Cell.

[12] Lee HCCS, Choudhary S, Aalto AP, Maiti M, Bamford DH, Liu Y (2009). qiRNA is a new type of small interfering RNA induced by DNA damage. Nature.

[13] Brosnan CA, Voinnet O (2009). The long and the short of noncoding RNAs. Curr Opin Cell Biol.

[14] Wei W, Ba Z, Gao M, Wu Y, Ma Y, Amiard S, White CI, Rendtlew Danielsen JM, Yang YG, Qi Y (2012). A role for small RNAs in DNA double-strand break repair. Cell.

[15] Mochizuki K, Fine NA, Fujisawa T, Gorovsky MA (2002). Analysis of a piwi-related gene implicates small RNAs in genome rearrangement in tetrahymena. Cell.

[16] Le Thomas A, Fejes Tòth K, Aravin AA (2014). To be or not to be a piRNA: genomic origin and processing of piRNAs. Genome Biol.

[17] Aravin AA, Bourc’his D (2008). Small RNA guides for de novo DNA methylation in mammalian germ cells. Genes Dev.

[18] Kuramochi-Miyagawa S, Watanabe T, Gotoh K, Takamatsu K, Chuma S, Kojima-Kita K, Shiromoto Y, Asada N, Toyoda A, Fujiyama A, Totoki Y, Shibata T, Kimura T, Nakatsuji N, Noce T, Sasaki H, Nakano T (2010). MVH in piRNA processing and gene silencing of retrotransposons. Genes Dev.

[19] Haag JR, Pikaard CS (2011). Multisubunit RNA polymerases IV and V: purveyors of non-coding RNA for plant gene silencing. Nat Rev Mol Cell Biol.

[20] Ender C, Krek A, Friedlander MR, Beitzinger M, Weinmann L, Chen W, Pfeffer S, Rajewsky N, Meister G (2008). A human snoRNA with microRNA-like functions. Mol Cell.

[21] Melamed Z, Levy A, Ashwal-Fluss R, Lev-Maor G, Mekahel K, Atias N, Gilad S, Sharan R, Levy C, Kadener S, Ast G (2013). Alternative splicing regulates biogenesis of miRNAs located across exon-intron junctions. Mol Cell.

[22] Moustafa A, Beszteri B, Maier UG, Bowler C, Valentin K, Bhattacharya D (2009). Genomic footprints of a cryptic plastid endosymbiosis in diatoms. Science.

[23] Bowler C, Allen AE, Badger JH, Grimwood J, Jabbari K, Kuo A, Maheswari U, Martens C, Maumus F, Otillar RP, Rayko E, Salamov A, Vandepoele K, Beszteri B, Gruber A, Heijde M, Katinka M, Mock T, Valentin K, Verret F, Berges JA, Brownlee C, Cadoret JP, Chiovitti A, Choi CJ, Coesel S, De Martino A, Detter JC, Durkin C, Falciatore A (2008). The Phaeodactylum genome reveals the evolutionary history of diatom genomes. Nature.

[24] Armbrust EV, Berges JA, Bowler C, Green BR, Martinez D, Putnam NH, Zhou S, Allen AE, Apt KE, Bechner M, Brzezinski MA, Chaal BK, Chiovitti A, Davis AK, Demarest MS, Detter JC, Glavina T, Goodstein D, Hadi MZ, Hellsten U, Hildebrand M, Jenkins BD, Jurka J, Kapitonov VV, Kroger N, Lau WW, Lane TW, Larimer FW, Lippmeier JC, Lucas S (2004). The genome of the diatom Thalassiosira pseudonana: ecology, evolution, and metabolism. Science.

[25] Maumus F, Allen AE, Mhiri C, Hu H, Jabbari K, Vardi A, Grandbastien MA, Bowler C (2009). Potential impact of stress activated retrotransposons on genome evolution in a marine diatom. BMC Genomics.

[26] Veluchamy A, Lin X, Maumus F, Rivarola M, Bhavsar J, Creasy T, O’Brien K, Sengamalay NA, Tallon LJ, Smith AD, Rayko E, Ahmed I, Le Crom S, Farrant GK, Sgro JY, Olson SA, Bondurant SS, Allen A, Rabinowicz PD, Sussman MR, Bowler C, Tirichine L (2013). Insights into the role of DNA methylation in diatoms by genome-wide profiling in Phaeodactylum tricornutum. Nat Commun.

[27] Norden-Krichmar TM, Allen AE, Gaasterland T, Hildebrand M (2011). Characterization of the small RNA transcriptome of the diatom, Thalassiosira pseudonana. PLoS One.

[28] Huang A, He L, Wang G (2011). Identification and characterization of microRNAs from Phaeodactylum tricornutum by high-throughput sequencing and bioinformatics analysis. BMC Genomics.

[29] Mathelier A, Carbone A (2010). MIReNA: finding microRNAs with high accuracy and no learning at genome scale and from deep sequencing data. Bioinformatics.

[30] De Riso V, Raniello R, Maumus F, Rogato A, Bowler C, Falciatore A (2009). Gene silencing in the marine diatom Phaeodactylum tricornutum. Nucleic Acids Res.

[31] Depauw FA, Rogato A, Ribera D’Alcala M, Falciatore A (2012). Exploring the molecular basis of responses to light in marine diatoms. J Exp Bot.

[32] Martin JHC, Coale KH, Johnson KS, Fitzwater SE, Gordon RM, Tanner SJ, Hunter CN, Elrod VA, Nowicki JL, Coley TL, Barber RT, Lindley S, Watson AJ, van Scoy K, Law CS, Liddicoat MI, Ling R, Stanton T, Stockel J, Collins C, Anderson A, Bidigare R, Ondrusek M, Latasa M, Millero FJ, Lee K, Yao W, Zhang JZ, Friederich G, Sakamoto C (1994). Testing the iron hypothesis in ecosystems of the equatorial Pacific Ocean. Nature.

[33] Boyd PW (2007). Biogeochemistry: iron findings. Nature.

[34] Axtell MJ (2013). Classification and comparison of small RNAs from plants. Annu Rev Plant Biol.

[35] Meyers BC, Axtell MJ, Bartel B, Bartel DP, Baulcombe D, Bowman JL, Cao X, Carrington JC, Chen X, Green PJ, Griffiths-Jones S, Jacobsen SE, Mallory AC, Martienssen RA, Poethig RS, Qi Y, Vaucheret H, Voinnet O, Watanabe Y, Weigel D, Zhu JK (2008). Criteria for annotation of plant MicroRNAs. Plant Cell.

[36] Li Y, Zhang Z, Liu F, Vongsangnak W, Jing Q, Shen B (2012). Performance comparison and evaluation of software tools for microRNA deep-sequencing data analysis. Nucleic Acids Res.

[37] Peng H, Shi J, Zhang Y, Zhang H, Liao S, Li W, Lei L, Han C, Ning L, Cao Y, Zhou Q, Chen Q, Duan E (2012). A novel class of tRNA-derived small RNAs extremely enriched in mature mouse sperm. Cell Res.

[38] Cole C, Sobala A, Lu C, Thatcher SR, Bowman A, Brown JW, Green PJ, Barton GJ, Hutvagner G (2009). Filtering of deep sequencing data reveals the existence of abundant Dicer-dependent small RNAs derived from tRNAs. RNA.

[39] Mathelier A, Carbone A (2013). Large scale chromosomal mapping of human microRNA structural clusters. Nucleic Acids Res.

[40] Macrae IJ, Zhou K, Li F, Repic A, Brooks AN, Cande WZ, Adams PD, Doudna JA (2006). Structural basis for double-stranded RNA processing by Dicer. Science.

[41] Kozomara A, Griffiths Jones S (2011). miRBase: integrating microRNA annotation and deep-sequencing data. Nucleic Acids Res.

[42] Roesser JR (2004). Both U2 snRNA and U12 snRNA are required for accurate splicing of exon 5 of the rat calcitonin/CGRP gene. RNA.

[43] Varkonyi-Gasic E, Wu R, Wood M, Walton EF, Hellens RP (2007). Protocol: a highly sensitive RT-PCR method for detection and quantification of microRNAs. Plant Methods.

[44] Babiarz JE, Ruby JG, Wang Y, Bartel DP, Blelloch R (2008). Mouse ES cells express endogenous shRNAs, siRNAs, and other Microprocessor-independent, Dicer-dependent small RNAs. Genes Dev.

[45] Couvillion MT, Sachidanandam R, Collins K (2010). A growth-essential Tetrahymena Piwi protein carries tRNA fragment cargo. Genes Dev.

[46] Loss-Morais G, Waterhouse PM, Margis R (2013). Description of plant tRNA-derived RNA fragments (tRFs) associated with argonaute and identification of their putative targets. Biol Direct.

[47] Nowacka M, Strozycki PM, Jackowiak P, Hojka-Osinska A, Szymanski M, Figlerowicz M (2013). Identification of stable, high copy number, medium-sized RNA degradation intermediates that accumulate in plants under non-stress conditions. Plant Mol Biol.

[48] Gebetsberger J, Polacek N (2013). Slicing tRNAs to boost functional ncRNA diversity. RNA Biol.

[49] Mortazavi A, Williams BA, McCue K, Schaeffer L, Wold B (2008). Mapping and quantifying mammalian transcriptomes by RNA-Seq. Nat Methods.

[50] Slotkin RK, Martienssen R (2007). Transposable elements and the epigenetic regulation of the genome. Nat Rev Genet.

[51] Maheswari U, Mock T, Armbrust EV, Bowler C (2009). Update of the Diatom EST Database: a new tool for digital transcriptomics. Nucleic Acids Res.

[52] Law JA, Jacobsen SE (2010). Establishing, maintaining and modifying DNA methylation patterns in plants and animals. Nat Rev Genet.

[53] Molnar A, Schwach F, Studholme DJ, Thuenemann EC, Baulcombe DC (2007). miRNAs control gene expression in the single-cell alga Chlamydomonas reinhardtii. Nature.

[54] Liang C, Zhang X, Zou J, Xu D, Su F, Ye N (2010). Identification of miRNA from Porphyra yezoensis by high-throughput sequencing and bioinformatics analysis. PLoS One.

[55] Cock JM, Sterck L, Rouze P, Scornet D, Allen AE, Amoutzias G, Anthouard V, Artiguenave F, Aury JM, Badger JH, Beszteri B, Billiau K, Bonnet E, Bothwell JH, Bowler C, Boyen C, Brownlee C, Carrano CJ, Charrier B, Cho GY, Coelho SM, Collen J, Corre E, Da Silva C, Delage L, Delaroque N, Dittami SM, Doulbeau S, Elias M, Farnham G (2010). The Ectocarpus genome and the independent evolution of multicellularity in brown algae. Nature.

[56] Billoud B, Nehr Z, Le Bail A, Charrier B (2014). Computational prediction and experimental validation of microRNAs in the brown alga Ectocarpus siliculosus. Nucleic Acids Res.

[57] Gross J, Wajid S, Price DC, Zelzion E, Li J, Chan CX, Bhattacharya D (2013). Evidence for widespread exonic small RNAs in the glaucophyte alga Cyanophora paradoxa. PLoS One.

[58] Cerutti H, Ma X, Msanne J, Repas T (2011). RNA-mediated silencing in Algae: biological roles and tools for analysis of gene function. Eukaryot Cell.

[59] Cheloufi S, Dos Santos CO, Chong MM, Hannon GJ (2010). A dicer-independent miRNA biogenesis pathway that requires Ago catalysis. Nature.

[60] Dueck A, Meister G (2010). MicroRNA processing without Dicer. Genome Biol.

[61] Lee Y, Kim M, Han J, Yeom KH, Lee S, Baek SH, Kim VN (2004). MicroRNA genes are transcribed by RNA polymerase II. EMBO J.

[62] Wahl MC, Will CL, Luhrmann R (2009). The spliceosome: design principles of a dynamic RNP machine. Cell.

[63] Berg MG, Singh LN, Younis I, Liu Q, Pinto AM, Kaida D, Zhang Z, Cho S, Sherrill-Mix S, Wan L, Dreyfuss G (2012). U1 snRNP determines mRNA length and regulates isoform expression. Cell.

[64] Allen AE, Laroche J, Maheswari U, Lommer M, Schauer N, Lopez PJ, Finazzi G, Fernie AR, Bowler C (2008). Whole-cell response of the pennate diatom Phaeodactylum tricornutum to iron starvation. Proc Natl Acad Sci U S A.

[65] Lommer M, Specht M, Roy AS, Kraemer L, Andreson R, Gutowska MA, Wolf J, Bergner SV, Schilhabel MB, Klostermeier UC Beiko RG, Rosenstiel P, Hippler M, Laroche J (2012). Genome and low-iron response of an oceanic diatom adapted to chronic iron limitation. Genome Biol.

[66] Sobala A, Hutvagner G (2011). Transfer RNA-derived fragments: origins, processing, and functions. Wiley Interdiscip Rev RNA.

[67] Franzen O, Arner E, Ferella M, Nilsson D, Respuela P, Carninci P, Hayashizaki Y, Aslund L, Andersson B, Daub CO (2011). The short non-coding transcriptome of the protozoan parasite Trypanosoma cruzi. PLoS Negl Trop Dis.

[68] Thompson DM, Lu C, Green PJ, Parker R (2008). tRNA cleavage is a conserved response to oxidative stress in eukaryotes. RNA.

[69] Garcia-Silva MR, Frugier M, Tosar JP, Correa-Dominguez A, Ronalte-Alves L, Parodi-Talice A, Rovira C, Robello C, Goldenberg S, Cayota A (2010). A population of tRNA-derived small RNAs is actively produced in Trypanosoma cruzi and recruited to specific cytoplasmic granules. Mol Biochem Parasitol.

[70] Rebollo R, Romanish MT, Mager DL (2012). Transposable elements: an abundant and natural source of regulatory sequences for host genes. Annu Rev Genet.

[71] Fedoroff NV (2012). Presidential address. Transposable elements, epigenetics, and genome evolution. Science.

[72] Mari-Ordonez A, Marchais A, Etcheverry M, Martin A, Colot V, Voinnet O (2013). Reconstructing de novo silencing of an active plant retrotransposon. Nat Genet.

[73] Kulis M, Queiros AC, Beekman R, Martin-Subero JI (1829). Intragenic DNA methylation in transcriptional regulation, normal differentiation and cancer. Biochim Biophys Acta.

[74] Teixeira FK, Colot V (2010). Repeat elements and the Arabidopsis DNA methylation landscape. Heredity (Edinb).

[75] Qian W, Miki D, Zhang H, Liu Y, Zhang X, Tang K, Kan Y, La H, Li X, Li S, Zhu X, Shi X, Zhang K, Pontes O, Chen X, Liu R, Gong Z, Zhu J-K (2012). A histone acetyltransferase regulates active DNA demethylation in Arabidopsis. Science.

[76] Lei J, Levin SA, Nie Q (2014). Mathematical model of adult stem cell regeneration with cross-talk between genetic and epigenetic regulation. Proc Natl Acad Sci U S A.

[77] Huff JT, Zilberman D (2014). Dnmt1-independent CG methylation contributes to nucleosome positioning in diverse eukaryotes. Cell.

[78] Guillard RRL, Smith WL, Chanley MH (1975). Culture of phytoplankton for feeding marine invertebrates. Culture of Marine Invertebrate Animals USA: New York.

[79] Emde AK, Grunert M, Weese D, Reinert K, Sperling SR (2010). MicroRazerS: rapid alignment of small RNA reads. Bioinformatics.

[80] Quinlan AR, Hall IM (2010). BEDTools: a flexible suite of utilities for comparing genomic features. Bioinformatics.

[81] Dale RK, Pedersen BS, Quinlan AR (2011). Pybedtools: a flexible Python library for manipulating genomic datasets and annotations. Bioinformatics.

[82] Gardner PP, Daub J, Tate J, Moore BL, Osuch IH, Griffiths-Jones S, Finn RD, Nawrocki EP, Kolbe DL, Eddy SR, Bateman A (2011). Rfam: Wikipedia, clans and the “decimal” release. Nucleic Acids Res.

[83] Lowe TM, Eddy SR (1997). tRNAscan-SE: a program for improved detection of transfer RNA genes in genomic sequence. Nucleic Acids Res.

[84] Kielbasa SM, Wan R, Sato K, Horton P, Frith MC (2011). Adaptive seeds tame genomic sequence comparison. Genome Res.

[85] Hofacker IL (2003). Vienna RNA secondary structure server. Nucleic Acids Res.

[86] Darty K, Denise A, Ponty Y (2009). VARNA: Interactive drawing and editing of the RNA secondary structure. Bioinformatics.

[87] Moxon S, Schwach F, Dalmay T, Maclean D, Studholme DJ, Moulton V (2008). A toolkit for analysing large-scale plant small RNA datasets. Bioinformatics.

[88] Hendrix D, Levine M, Shi W (2010). miRTRAP, a computational method for the systematic identification of miRNAs from high throughput sequencing data. Genome Biol.

[89] Schwach F, Moxon S, Moulton V, Dalmay T (2009). Deciphering the diversity of small RNAs in plants: the long and short of it. Brief Funct Genomic Proteomic.

[90] Chen C, Ridzon DA, Broomer AJ, Zhou Z, Lee DH, Nguyen JT, Barbisin M, Xu NL, Mahuvakar VR, Andersen MR, Lao KQ, Livak KJ, Guegler KJ (2005). Real-time quantification of microRNAs by stem-loop RT-PCR. Nucleic Acids Res.

[91] Tang F, Hajkova P, Barton SC, Lao K, Surani MA (2006). MicroRNA expression profiling of single whole embryonic stem cells. Nucleic Acids Res.

[92] Coesel S, Mangogna M, Ishikawa T, Heijde M, Rogato A, Finazzi G, Todo T, Bowler C, Falciatore A (2009). Diatom PtCPF1 is a new cryptochrome/photolyase family member with DNA repair and transcription regulation activity. EMBO Rep.

[93] Falciatore A, Casotti R, Leblanc C, Abrescia C, Bowler C (1999). Transformation of Nonselectable Reporter Genes in Marine Diatoms. Mar Biotechnol (NY).

