# Supplementary material for: The diversity of small non-coding RNAs in the diatom Phaeodactylum tricornutum
Source: BMC Genomics. 2014 Aug 20;15(1):698. doi: 10.1186/1471-2164-15-698 (PMC4247016; doi:10.1186/1471-2164-15-698)
Supplement: Supplementary file 1 — Additional file 1: Table S1: Summary of the properties of all potential sRNA producing loci. (PDF 42 KB) [file 12864_2014_6681_MOESM1_ESM.pdf]

Additional Table S1

Note	Overlapping regions (>10%)						number of regions	mean region width	% unique reads in NL	cum. % unique reads in NL	cum. % of all regions width	median RPKM in NL	mean RPKM unique in NL	median RPKM multi in NL	mean RPKM multi in NL	count unique in NL	count multi in NL	Total width (bp)
	Transposons	Genes	Highly Meth. Regions	Candidate Loci: tRNA (28)	Candidate Loci: ncRNA (2)	Candidate Loci: other candidates (20)												
Transposons assocated with methylation make up 56% of the unique reads. median RPKM is >10	x	-	x	-	-	-	359	4303	0.56	0.56	6%	10	98	62	243	325892	737121	1544645
Nine of our 20 candidate regions concentrate 17.6% of the fragments	-	-	-	-	-	x	9	353	0.176	0.736	6%	1249	42122	1249	42122	102332	102332	3175
HMR not overlapping genes nor TEs	-	-	x	-	-	-	180	2196	0.078	0.814	7%	4	38	30	165	45609	174361	395237
HMRs overlapping genes	-	x	x	-	-	-	78	1196	0.044	0.858	7%	4	79	17	129	25608	26493	93260
HMR overlapping TEs and genes	x	x	x	-	-	-	18	4873	0.042	0.9	8%	15	123	92	187	24576	32498	87712
tRNAs and ncRNAs	-	-	-	x	-	-	18	320	0.02	0.92	8%	718	1824	1318	2968	11481	16122	5762
	-	-	-	-	x	-	2	260	0.011	0.931	8%	11213	11213	11373	11373	6190	6291	520
	-	x	-	x	-	-	8	565	0.004	0.935	8%	334	528	1320	2230	2415	11083	4516
Proportion of fragments explained by the categories described above: 93.5%																		
Those two lines are characteristics of degradation products: short regions with low coverage (median RPKM =0)	-	x	-	-	-	-	4852	170	0.034	0.969	11%	0	21	13	66	19532	24393	822432
	-	-	-	-	-	-	2849	177	0.024	0.993	13%	0	24	12	77	14206	23137	505188
	x	-	-	-	-	-	261	504	0.003	0.996	13%	0	16	13	37	1809	5365	131475
	-	x	-	-	-	x	8	291	0.003	0.999	13%	472	712	472	712	1807	1807	2326
	x	x	-	-	-	-	22	485	0	0.999	13%	3	5	12	13	58	158	10669
	x	-	-	x	-	-	1	318	0	0.999	13%	42	42	1246	1246	15	448	318

Analysis of Periods

HMR regions (351 regions) (**period median: 180bp**)

	First period by FFT		
overlap	<160bp	160-200 bp	>200 bp
ov. TE	40	43	27
no ov TE	86	87	68
