# Supplementary material for: The diversity of small non-coding RNAs in the diatom Phaeodactylum tricornutum
Source: BMC Genomics. 2014 Aug 20;15(1):698. doi: 10.1186/1471-2164-15-698 (PMC4247016; doi:10.1186/1471-2164-15-698)
Supplement: Supplementary file 2 — Additional file 2: Table S2: List and properties of regions annotated with our set of filters for miRNAs identification. (PDF 44 KB) [file 12864_2014_6681_MOESM2_ESM.pdf]

Additional Table S2

Candidate region						Summary of evidences		# libraries detected		Predictions with MIReNA			
chr	start	end	nreads in region	width	Annotation	Lib.	MiReNA	Our study (5)	Huang et al 2011 (3)	# of stems	MIRENA precursor length	MFE	empirical pvalue*
chr_2	28 963	29 125	561672	163	intergenic	Y	N	5	3				
chr_30	79 190	79 356	14975	167	intergenic	Y	N	2	3				
chr_5	280 626	280 888	5153	263	intergenic	N	N	2	0				
chr_1	1 306 372	1 306 458	3472	87	intergenic	N	N	1	0				
chr_10	426 924	427 420	3054	497	intergenic	Y	Y	3	0	<=3	151	-77.2	**0.004**
chr_10	360 059	360 290	2327	232	intergenic	N	N	1	0				
chr_21	188 517	188 797	1648	281	intergenic	N	N	2	1				
chr_10	498 075	498 320	1575	246	intron	Y	Y	3	0	<=3	127	-43.3	0.362
chr_9	914 835	914 924	1285	90	intergenic	N	N	1	0				
chr_4	1 285 744	1 285 881	1222	138	exon	N	N	1	0				
chr_4	945 389	945 546	787	158	intron	N	N	2	0				
chr_24	152 153	152 280	762	128	intron	N	N	1	0				
chr_21	509 377	509 782	645	406	exon	Y	N	3	0				
chr_29	277 384	277 549	552	166	intergenic	N	Y	1	0	<=3	139	-58.9	0.057
chr_28	260 020	260 229	544	210	intergenic	Y	Y	1	3	<=3	176	-59.3	0.490
chr_13	260 086	260 248	482	163	intron	N	N	1	1				
chr_1	1 044 424	1 044 453	434	30	intron	N	N	1	0				
chr_1	2 453 906	2 454 049	406	144	intergenic	N	Y	1	0	<=3	63	-37.5	**0.001**
chr_1	1 804 606	1 804 753	362	148	intron	N	Y	1	1	<=3	221	-81.0	0.335
chr_11	607 137	607 236	319	100	exon	N	N	1	0				

*: computed by drawing 1000 regions of the same length and recording each time the MFE.
