# Supplementary material for: The diversity of small non-coding RNAs in the diatom Phaeodactylum tricornutum
Source: BMC Genomics. 2014 Aug 20;15(1):698. doi: 10.1186/1471-2164-15-698 (PMC4247016; doi:10.1186/1471-2164-15-698)

Additional Figure S1

chr10:426.924-427.420

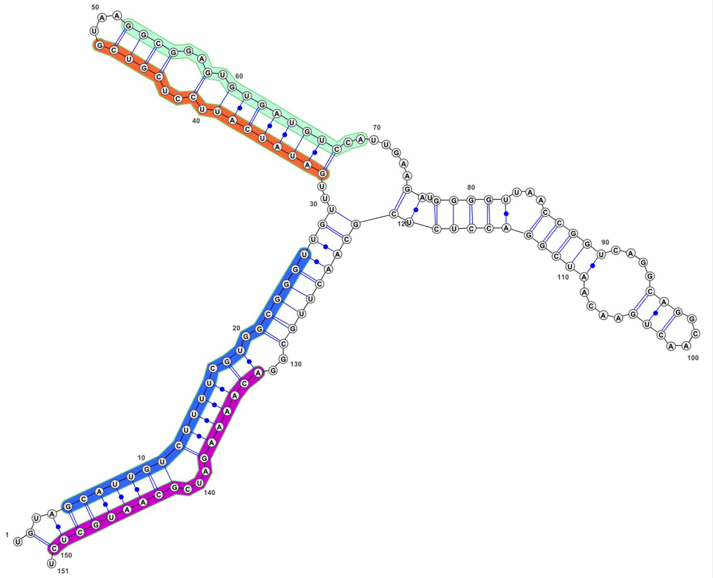

- detected in 3 of our libraries.
- predicted with a miRNA structure by MiReNA with the mature sequence colored in violet ( ).
- Two stem loops structures (with the mature sequences in the violet and green:) are compatible with read profiles.
- The green sequence is mainly present in –Fe condition.

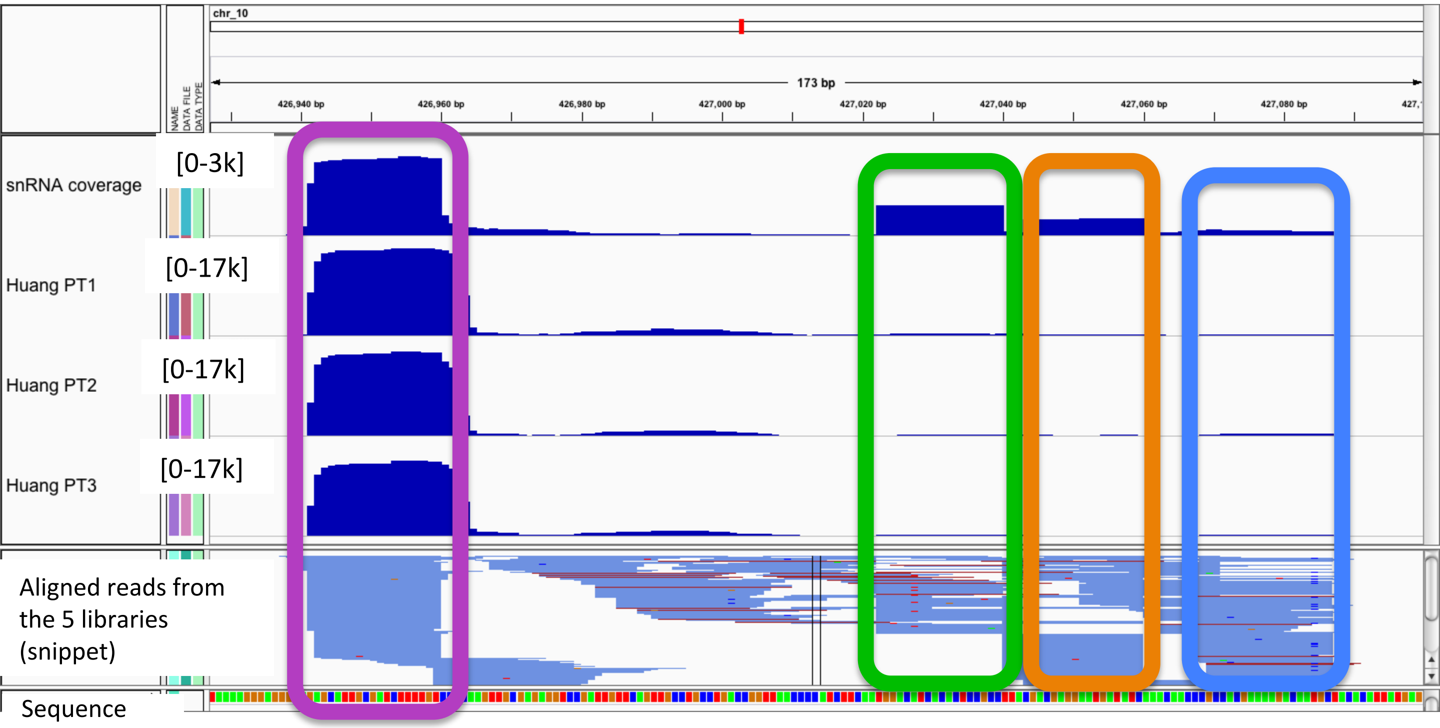

Supplement: Supplementary file 3 — Additional file 3: Figure S1: Predicted secondary structures and read profiles for a miRNA-like candidate on chr10 predicted by MIReNA. (PDF 295 KB) [file 12864_2014_6681_MOESM3_ESM.pdf]
