# Supplementary material for: The diversity of small non-coding RNAs in the diatom Phaeodactylum tricornutum
Source: BMC Genomics. 2014 Aug 20;15(1):698. doi: 10.1186/1471-2164-15-698 (PMC4247016; doi:10.1186/1471-2164-15-698)

Additional Figure S2

chr1:2.453.906-2.454.049

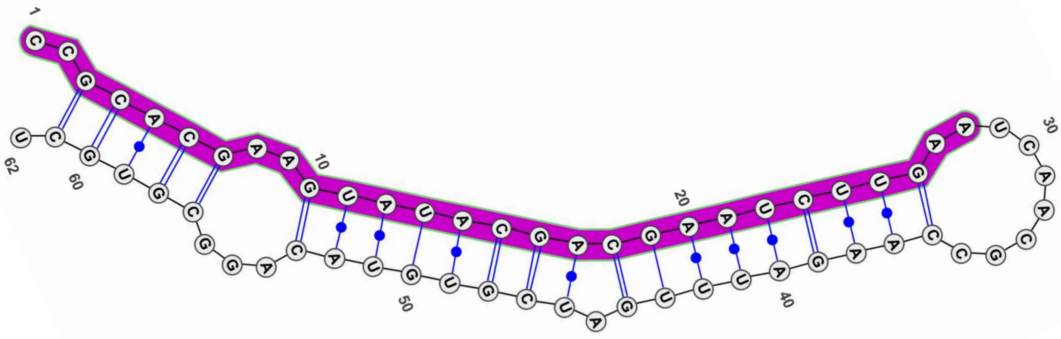

- Detected in 1 library with lower coverage
- Significantly predicted miRNA-like structure, with a stem loop compatible with the reads profile
- No star sequence is detected

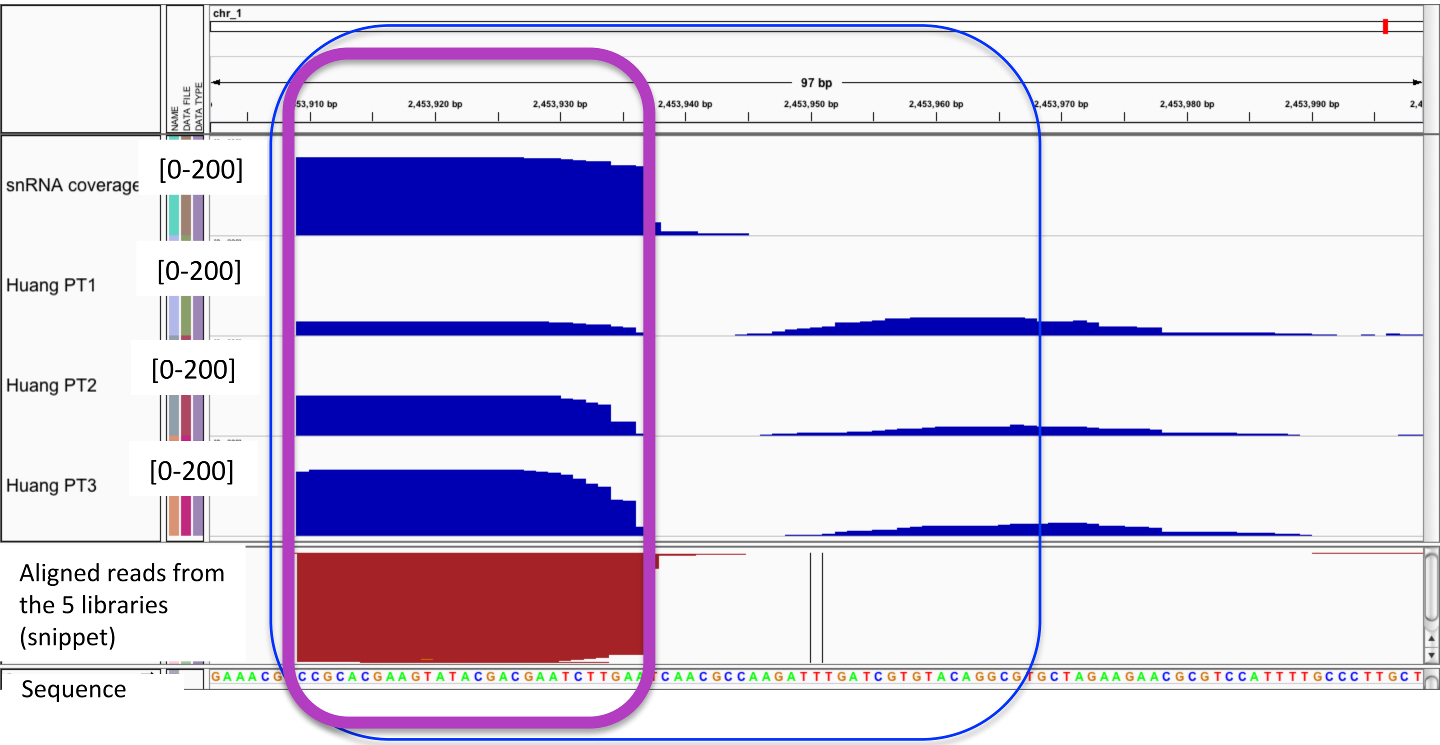

Supplement: Supplementary file 4 — Additional file 4: Figure S2: Predicted secondary structures and read profiles for a miRNA-like candidate on chr1 predicted by MIReNA. (PDF 292 KB) [file 12864_2014_6681_MOESM4_ESM.pdf]
