# Supplementary material for: The diversity of small non-coding RNAs in the diatom Phaeodactylum tricornutum
Source: BMC Genomics. 2014 Aug 20;15(1):698. doi: 10.1186/1471-2164-15-698 (PMC4247016; doi:10.1186/1471-2164-15-698)
Supplement: Supplementary file 5 — Additional file 5: Supplementary data and methods. (DOCX 58 KB) [file 12864_2014_6681_MOESM5_ESM.docx]

# Additional file 5

# SUPPLEMENTARY DATA and METHODS

**Read mapping parameters and quality control**

MicroRazer was run with parameters -sE -m 100 -pa. This mapping strategy anchors each read on its 5' end with a 16 bp seed (allowing up to one mismatch on the seed region and with a minimum of 90% sequence identity), and reports the set of all maximal matches.

In agreement with the library preparation, all reads that aligned on less than 18nt were discarded. Additionally, we discarded reads that would be shorter than 18nt after trimming adenosines from their 3’ end after alignment. This was done to avoid sequences that would align in a A-rich region.

**Filtering steps used in the annotation workflow**

We first used all reads with at least one mapping position along the genome and defined sRNA producing loci by grouping sequences distant by less than 100bp from each other. Only loci with at least 10 reads were considered. With this definition we could identify 8665 sRNA producing loci.

Over the identified sRNA producing loci, we selected candidates with sRNA processing profiles by keeping only those that fulfilled the following criteria:

- low sequences diversity (number of reads > 2x number of sequences)
- strand specificity (reads on one strand > 2x reads on the other strand)
- one over-represented sequence (most abundant sequence > 25% of all the reads)
- few degradation products, that is, half of the reads should have an aligned length comprised between 19nt and 29nt.
- the region should be shorter than 1kb, and the most abundant sequence should have at least 100 RPM (Reads Per Million).
- the region should not overlap a repeat region.

A set of resulting regions was obtained for each library. Then, all regions have been merged across the 5 different libraries by taking the union of all genomic intervals.

**Results of predicting sRNAs on the independent library from Huang *et al***. [[1](#_ENREF_1)]

We observed a good agreement with the intial set of 50 regions (Figure 1A) that we predicted when using our filter on the 3 independent libraries from Huang et al., recovering a total of 23 regions. Of those, we detected 16 tRNA-related sRNAs, and one candidate that overlaps the 5S rRNA (Figure 1). Among the other candidates, the ones on chr2:28.963-29.125, chr30:79.190-79.356 and chr28:260.020-260.229 are recovered in all libraries.

We should also note that the authors predicted 13 miRNAs by selecting the regions that could fold in a stem-loop structure around every sequence in their three libraries but failed to provide experimental evidence for any of the predicted mature sequences. None of our 50 candidate regions overlapped with any of these 13 predicted miRNAs. These discrepancies can be explained by the fact that each of their putative miRNAs is supported by a too low number of reads (10 out of 13 cases) or has an insufficient relative contribution to the candidate region (5 out of 13 cases). In some cases, (e. g. pti-miR5473 and pti-miR5476) the predicted precursors are located in the middle of a repeat element, questioning the robustness of the prediction.

**References**

1. Huang A, He L, Wang G: **Identification and characterization of microRNAs from Phaeodactylum tricornutum by high-throughput sequencing and bioinformatics analysis.** *BMC Genomics* 2011, **12:**337.

2. Norden-Krichmar TM, Allen AE, Gaasterland T, Hildebrand M: **Characterization of the small RNA transcriptome of the diatom, Thalassiosira pseudonana.** *PLoS One* 2011, **6:**e22870.

3. Mathelier A, Carbone, A.: **MIReNA: finding microRNAs with high accuracy and no learning at genome scale and from deep sequencing data.** *Bioinformatics* 2010, **26:**2226-2234.

#

# Additional Figures and Tables Legends:

**Additional Figures S1 and S2: Predicted secondary structures and read profiles for the two miRNA-like candidates predicted by MIReNA [3].**

The most abundant fragments are highlighted on the predicted RNA secondary structure and their genomic location framed with the same color on the screenshot of the reads profile. The reads coverages are reported for (from top to bottom): our 5 libraries pooled, and Huang libraries PT1, PT2 and PT3. A snippet of aligned reads in our 5 libraries is reported on the bottom.

**Additional Figure S3: small RNA fragments expressed in *T. pseudonana.***

Analysis of the smallRNA sequences published by Norden-Krichmar *et al.* [[2](#_ENREF_2)] after alignment to *T. pseudonana* genome (using the same mapping parameters as described in the methods).

(A) Screenshot of the reads profile that matched significantly to the *P. tricornutum* candidate located in intergenic regions of chromosome 2 (chr2:29.039-29.123). The alignment between the *P. tricornutum* mature fragment and the *T. pseudonana* fragment is reported below.

(B) Screenshot of the reads profile on the region surrounding the *T. pseudonana* U2 snRNA. The U2-3’ fragment is highlighted with a green arrow.

(C) Fragment length distribution of reads that cover tRNA genes for the two biological replicates reported in Norden-Krichmar *et al*. [[2](#_ENREF_2)].

**Additional Figure S4: sRNA coverage and methylation level on genes.**

Comparison of methylation level and sRNA coverage on all coding genes (regions are defined to be the union of exons for a gene) that do not overlap transposable elements. The sRNA coverage is plotted for genes according to their methylation level.

**Additional Figure S5: Distribution of the main period in sRNA coverage on the set of HMR regions longer than 1000bp (351 regions).**

Histogram of the periods obtained with the highest spectrum after applying Fast Fourier transform on the read profiles to each of the Highly Methylated Regions longer than 1000bp.

**Additional Figure S6: Example of regions with periodic sRNA coverage detected.**

Screenshot of the reads profile on two different *P. tricornutum* genomic regions (A and B) where significant periods are detected by FFT analysis. For each 1000bp window slided along the region, a sine function is drawn over the experimental data when a period is detected. For each window, the corresponding period is reported on the x-axis in the middle of the window. Similar color intensities indicate similar periods. The annotation of genes and Transposable Elements in each region are indicated with blue rectangles below each plot.

**Additional Table S1: Summary of the properties of all potential sRNA producing loci**.

For each of the 8665 loci, we report coverage and size characteristics of regions according to the genomic annotation they overlap to: Transposons, Genes, Highly Methylated Regions and Candidate loci. The second table reports the analysis of period by Fast Fourier Transform (FFT) on the 351 HMR regions of more than 1000bp overlapping Transposable Elements (TE) or not.

**Additional Table S2: List and properties of regions annotated with our set of filters for miRNAs identification.**

For each candidate region we report:

1. Information about the candidate region: ID, coordinates in *P. tricornutum* genome (starting and ending positions), number of reads across the five conditions, region width, genomic location (intergenic, intron, exon);
2. Summary over the two types of evidence that were considered: agreement of the detection realized over multiple libraries (lib) - Y if detection was realized on more than half of the libraries either belonging to our sequencing data or to the Huang set [[1](#_ENREF_1)]; prediction result based on MIReNA [[3](#_ENREF_3)]
3. Number of libraries where the miRNA was detected, either across our five libraries or across the three libraries of Huang *et al*. [[1](#_ENREF_1)]
4. Properties of the predicted RNA secondary structures that were obtained by using MIReNA: number of stems on the structure, Minimum Folding Energy (MFE) and empirical pvalue.

The 2 candidates predicted as being miRNA-like (prediction by MIReNA [[3](#_ENREF_3)] and significant fold) are highlighted in grey.

**Additional Table S3: Properties of tRNA associated reads.**

For each anticodon we report:

1. coverage all experiments: the total effective counts within the tRNA genes corresponding to the anticodon.

2. PPM (prop per millions reads): the effective count in each condition normalized for 1 Millions reads aligned.

3. tRF pattern: the main localization of the reads within the tRNA genes are indicated.

4. length columns (18, 19, 20, 31-35): for each length range, we report for each condition, the proportion of reads observed within the tRNA.

Note: to take into account reads that can map within multiple locations, we use effective counts of the reads: we reweight their counts by the number of locations where it is equally aligned.

**Additional Table S4: List of primers utilized in the present work.**
