# Supplementary figures and images for: The diversity of small non-coding RNAs in the diatom Phaeodactylum tricornutum
Source: BMC Genomics. 2014 Aug 20;15(1):698. doi: 10.1186/1471-2164-15-698 (PMC4247016; doi:10.1186/1471-2164-15-698)

# Additional Figure S3

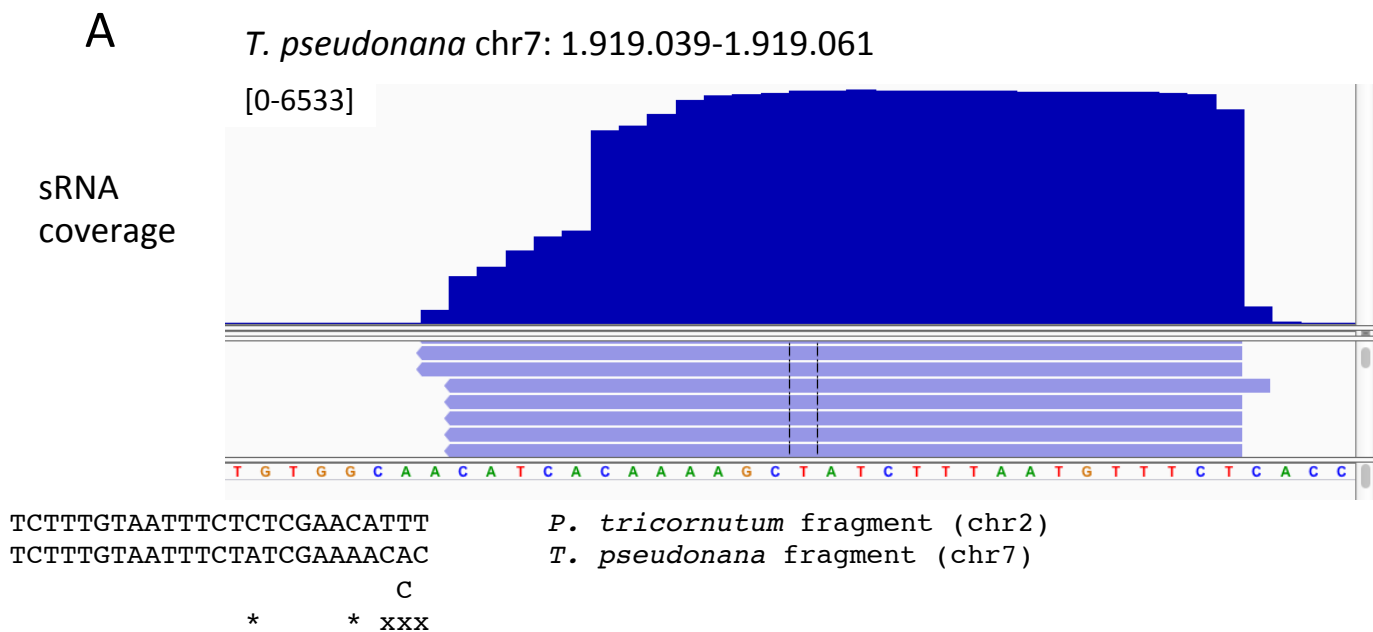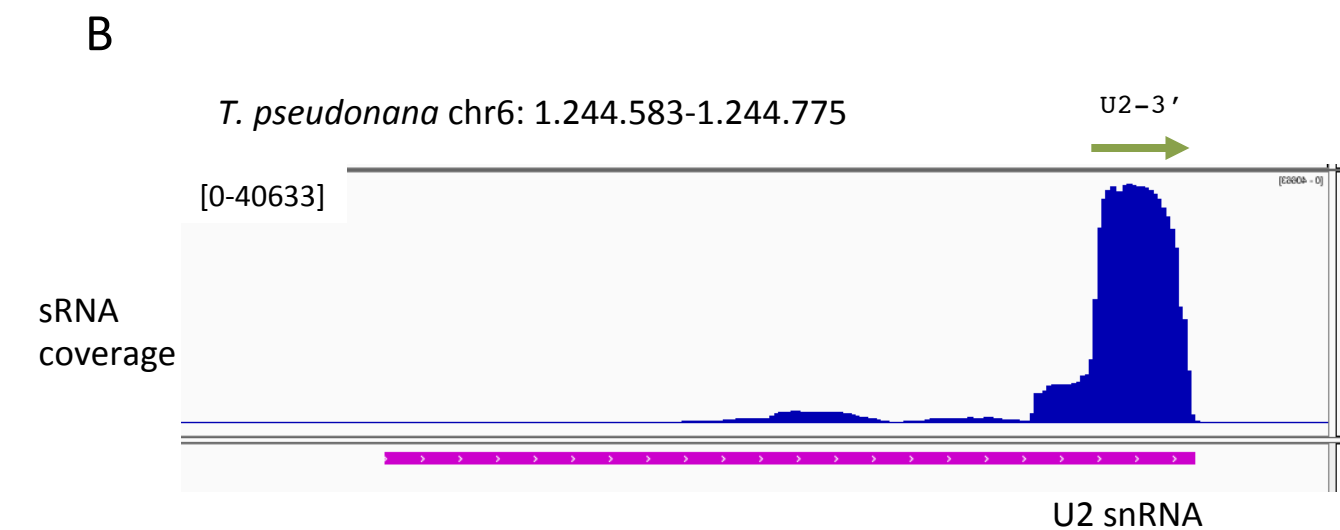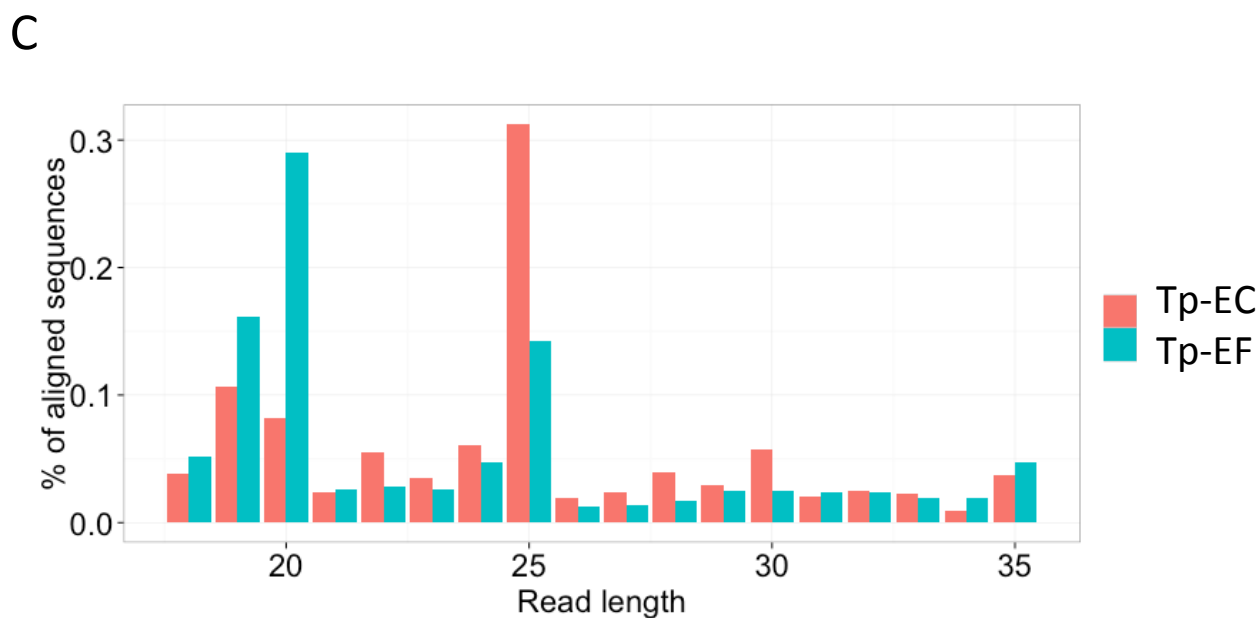

Supplement: Supplementary file 6 — Additional file 6: Figure S3: Small RNA fragments expressed in T. pseudonana. (PDF 158 KB) [file 12864_2014_6681_MOESM6_ESM.pdf]

Additional Figure S4

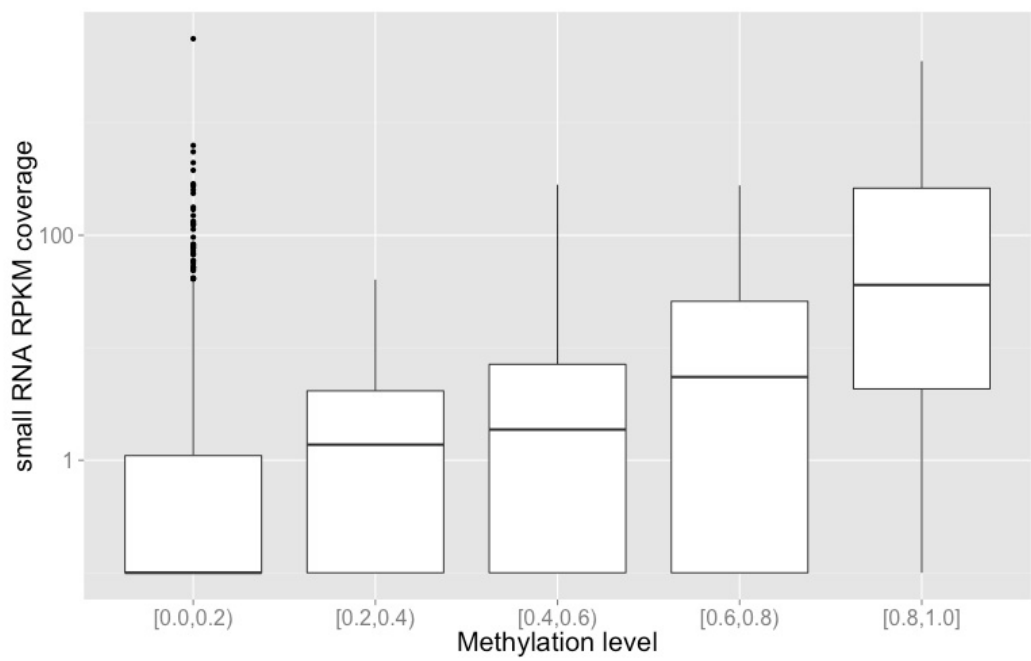

Supplement: Supplementary file 8 — Additional file 8: Figure S4: sRNA coverage and methylation level on genes. (PDF 57 KB) [file 12864_2014_6681_MOESM8_ESM.pdf]

Additional Figure S5

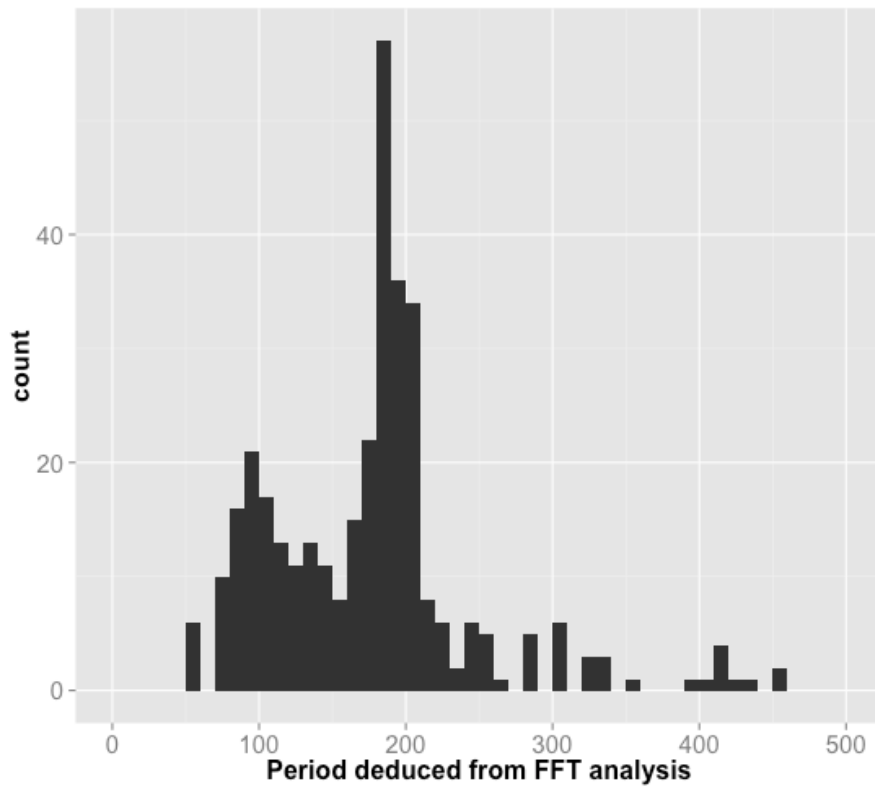

Supplement: Supplementary file 9 — Additional file 9: Figure S5: Distribution of the main period in sRNA coverage on the set of HMR regions longer than 1000 bp (351 regions). (PDF 41 KB) [file 12864_2014_6681_MOESM9_ESM.pdf]

# Additional Figure S6

A

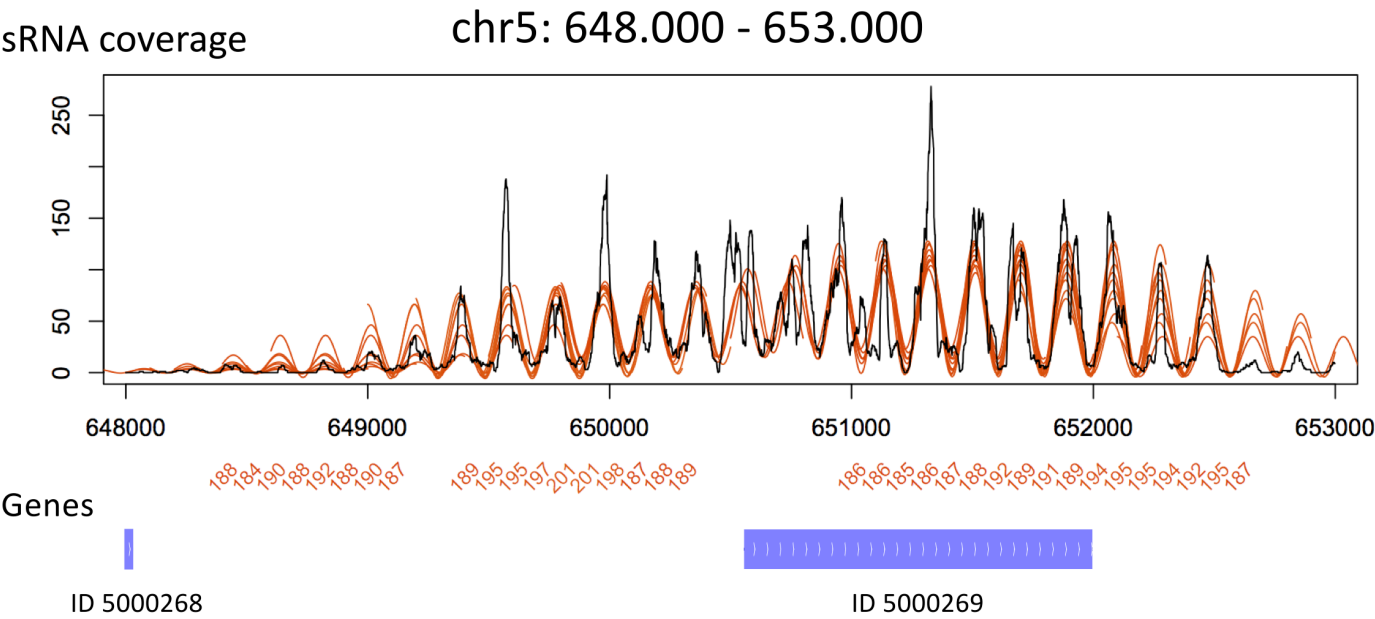

B

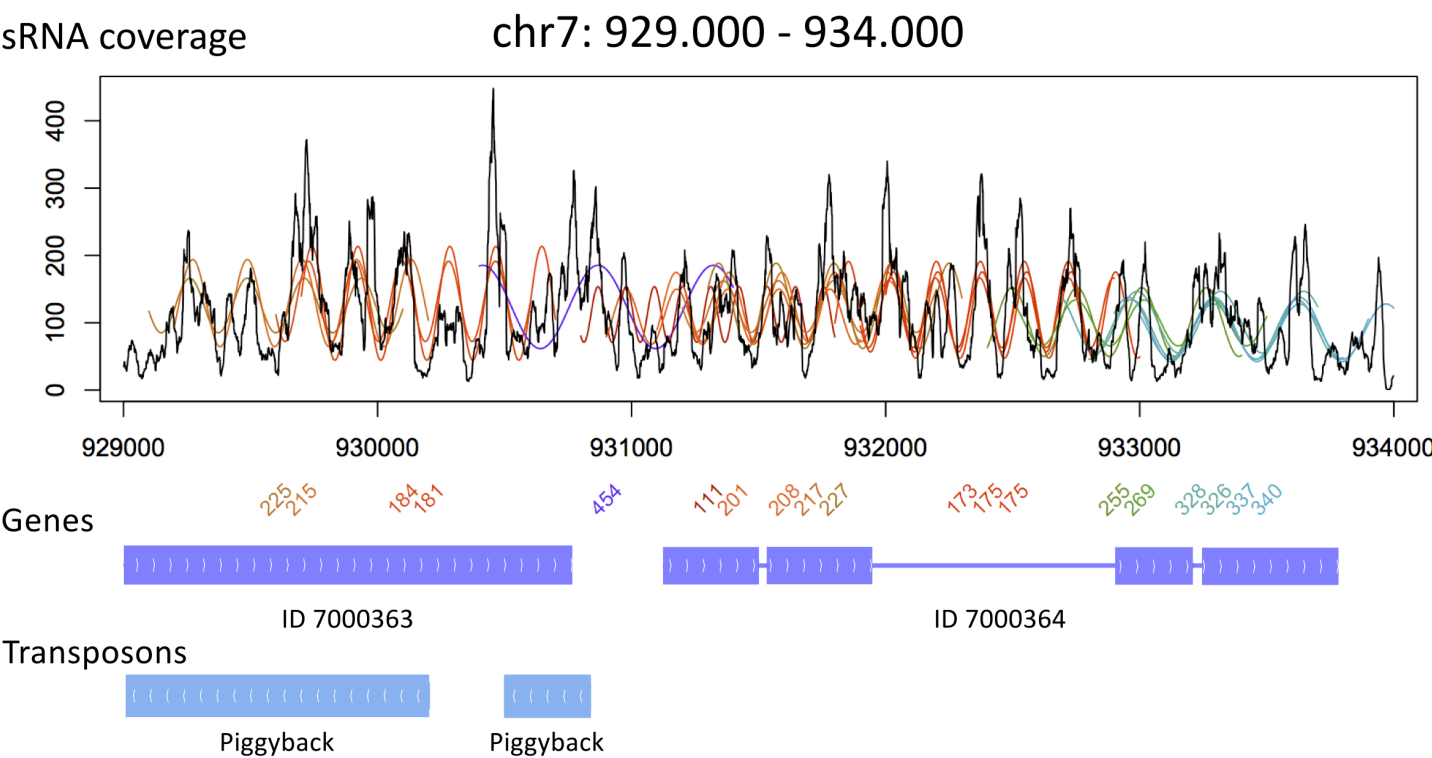

Supplement: Supplementary file 10 — Additional file 10: Figure S6: Example of regions with periodic sRNA coverage detected. (PDF 1 MB) [file 12864_2014_6681_MOESM10_ESM.pdf]
