# Supplementary material for: The diversity of small non-coding RNAs in the diatom Phaeodactylum tricornutum
Source: BMC Genomics. 2014 Aug 20;15(1):698. doi: 10.1186/1471-2164-15-698 (PMC4247016; doi:10.1186/1471-2164-15-698)
Supplement: Supplementary file 7 — Additional file 7: Table S3: Properties of tRNA associated reads. (PDF 66 KB) [file 12864_2014_6681_MOESM7_ESM.pdf]

Additional Table S3

			PPM (prop. per million reads)					tRF Pattern				length 18					Length 19					Length 20					Length 31-35				
Amino acid	Anticodon	Coverage all experiments	D	Fe	HL	LL	NL	5' Dloop-antiC	3' tRF short	3' tRFs long	5' tRF long	D	Fe	HL	LL	NL	D	Fe	HL	LL	NL	D	Fe	HL	LL	NL	D	Fe	HL	LL	NL
**Asp**	**GTC**	**54 006**	**2 542**	**25 213**	**10 336**	**5 761**	**3 548**		**Y**	**Y**		0.02	0.01	0.01	0.01	0.02	0.33	0.08	0.31	0.28	0.41	0.01	0	0	0.01	0	0.37	0.65	0.5	0.5	0.35
**Gly**	**GCC**	**18 351**	**792**	**5 142**	**7 526**	**1 351**	**3 671**		**Y**	**Fe**		0.04	0.01	0.01	0.01	0	0.36	0.13	0.77	0.57	0.72	0.03	0.02	0.04	0.03	0.03	0.23	0.54	0.08	0.19	0.13
Gly	TCC	15 474	896	1 915	7 055	1 659	4 325		Y	Fe		0.03	0.02	0.01	0.01	0	0.58	0.21	0.85	0.7	0.86	0.03	0.02	0.01	0.01	0.02	0.14	0.41	0.04	0.14	0.05
**Pro**	**AGG**	**17 736**	**858**	**6 100**	**6 873**	**1 357**	**2 350**		**Y**	**Fe, LL,HL**		0.02	0.01	0.01	0.02	0.01	0.03	0.02	0.01	0.02	0.03	0.49	0.2	0.72	0.58	0.7	0.22	0.58	0.14	0.25	0.14
Pro	CGG	10 745	398	2 114	6 078	1 456	1 374		Y			0.03	0.04	0.01	0.02	0.02	0.84	0.84	0.96	0.93	0.9	0.02	0.01	0	0.01	0.01	0.03	0.02	0	0	0.01
Pro	TGG	1 100	46	279	462	144	161		Y	Fe		0.06	0.07	0.04	0.04	0.06	0.5	0.69	0.86	0.75	0.8	0.09	0.02	0.02	0.04	0.04	0.06	0.05	0.02	0.05	0.02
Phe	GAA	10 703	1 804	1 159	2 287	1 703	2 309		Y			0	0.01	0.01	0	0.01	0.75	0.56	0.67	0.81	0.65	0.01	0.06	0.01	0.01	0.01	0	0	0	0	0
His	GTG	9 352	362	4 488	2 098	523	1 071		Y	Fe, LL		0	0.01	0.01	0	0	0.21	0.11	0.64	0.41	0.46	0.02	0.01	0.01	0.01	0.02	0.53	0.73	0.27	0.42	0.35
Val	AAC	6 258	590	1 254	2 165	955	921		Y	Fe		0.01	0.03	0.01	0.01	0.01	0.58	0.78	0.76	0.73	0.69	0.1	0.02	0.03	0.02	0.04	0.02	0.03	0	0	0.03
Val	CAC	4 790	245	1 214	1 958	691	583		Y	Fe		0.01	0.01	0	0	0.01	0.81	0.93	0.96	0.93	0.91	0.07	0.01	0.01	0.01	0.01	0.01	0.01	0.01	0	0.01
Val	TAC	473	71	75	96	101	54	Y	Y			0.19	0.17	0.03	0.04	0.03	0.2	0.1	0.05	0.03	0.07	0.14	0.36	0.61	0.65	0.48	0	0.03	0.04	0.01	0.02
**Glu**	**CTC**	**6 201**	**933**	**1 309**	**1 866**	**653**	**956**	**Y**			**Y**	0.05	0.05	0.04	0.05	0.04	0.1	0.05	0.07	0.06	0.08	0.06	0.02	0.04	0.06	0.05	0.24	0.48	0.24	0.23	0.25
Glu	TTC	2 928	274	1 443	489	185	153	Y	Y			0.49	0.63	0.49	0.52	0.4	0.26	0.21	0.2	0.18	0.27	0.18	0.03	0.08	0.05	0.09	0.05	0.07	0.1	0.07	0.07
Ala	AGC	5 423	397	1 176	1 014	840	1 298		Y	Fe		0.08	0.03	0.04	0.03	0.02	0.1	0.12	0.06	0.03	0.05	0.54	0.5	0.66	0.78	0.75	0.01	0.04	0	0.01	0.01
Ala	CGC	4 143	171	568	1 570	789	881		Y			0	0.02	0	0	0	0.52	0.54	0.91	0.86	0.77	0.07	0.05	0.01	0.01	0.03	0.04	0.07	0.01	0.01	0.01
Ala	TGC	1 061	62	489	254	101	50	Y			Fe	0.27	0.17	0.09	0.07	0.04	0.02	0.01	0.01	0.01	0.07	0.28	0.05	0.03	0.08	0.16	0.07	0.47	0.36	0.44	0.18
Gln	CTG	4 780	844	637	1 908	500	732		Y		Y	0.03	0.04	0.03	0.07	0.03	0.05	0.06	0.03	0.05	0.02	0.09	0.16	0.48	0.44	0.31	0.36	0.39	0.12	0.17	0.16
Gln	TTG	1 025	132	180	338	153	148	Y	Y			0.42	0.21	0.07	0.22	0.05	0.27	0.43	0.74	0.52	0.68	0.03	0.13	0.05	0.07	0.06	0.03	0.02	0.03	0.01	0.02
Ser	GCT	4 155	377	991	1 131	561	727	Y	Y	Fe		0.18	0.08	0.04	0.04	0.06	0.38	0.5	0.72	0.77	0.65	0.02	0.02	0.02	0.01	0.02	0.07	0.1	0.06	0.02	0.05
Ser	AGA	1 474	91	754	280	85	100	Y				0.22	0.5	0.15	0.15	0.09	0.27	0.27	0.43	0.44	0.55	0.13	0.02	0.06	0.07	0.13	0	0	0.01	0	0.01
Ser	CGA	900	95	145	335	162	111					0.2	0.29	0.1	0.1	0.06	0.18	0.23	0.11	0.08	0.07	0.07	0.05	0.35	0.15	0.26	0.01	0.02	0.01	0	0.07
Ser	TGA	478	52	170	94	59	40					0.49	0.37	0.14	0.23	0.29	0.14	0.35	0.62	0.45	0.49	0.03	0.01	0.07	0.01	0.04	0.02	0.01	0	0.01	0
Arg	TCG	3 892	194	1 157	1 528	357	613		Y	Fe, HL, NL		0	0	0	0.01	0.01	0.13	0.2	0.55	0.32	0.35	0.04	0.04	0.01	0.03	0.02	0.14	0.31	0.17	0.12	0.12
Arg	CCT	462	20	60	249	66	92					0.07	0.07	0.01	0.04	0.03	0.36	0.36	0.77	0.54	0.66	0.07	0.03	0.01	0.02	0.02	0	0.02	0.02	0.02	0.02
Arg	CCG	436	16	114	182	68	49					0.09	0.02	0.02	0.02	0.04	0.13	0.16	0.63	0.21	0.36	0.09	0.06	0.06	0.03	0.05	0	0.02	0.04	0.02	0
Arg	TCT	256	14	117	41	20	33					0	0.03	0.08	0.03	0.19	0	0.01	0	0.06	0	0	0.03	0.04	0.03	0.05	0.05	0.1	0.12	0.09	0.14
Thr	TGT	3 632	897	596	725	552	265	Y	Y		Y	0.13	0.06	0.04	0.06	0.05	0	0.02	0.02	0.02	0.04	0.62	0.19	0.35	0.4	0.48	0.11	0.54	0.35	0.25	0.1
Thr	AGT	1 717	139	366	405	249	380		Y		Fe	0.12	0.07	0.06	0.06	0.04	0.38	0.32	0.6	0.66	0.61	0.03	0.05	0.06	0.04	0.06	0.05	0.14	0.05	0.04	0.02
Thr	CGT	643	57	155	291	59	88		HL		Fe, HL	0.09	0.06	0.07	0.11	0.1	0.12	0.07	0.37	0.08	0.1	0.11	0.07	0.08	0.04	0.07	0.09	0.51	0.23	0.22	0.11
Leu	CAA	2 359	257	499	788	272	412	Y	Y			0.08	0.07	0.04	0.04	0.05	0.17	0.15	0.25	0.26	0.32	0.21	0.13	0.1	0.11	0.13	0	0.01	0.01	0.02	0
Leu	CAG	1 001	61	226	445	147	117					0.18	0.08	0.07	0.07	0.05	0.05	0.08	0.19	0.17	0.14	0.03	0.1	0.06	0.12	0.09	0.06	0.11	0.1	0.11	0.12
Leu	AAG	956	60	170	257	115	286		Y			0.47	0.34	0.08	0.03	0.02	0.02	0.12	0.03	0.02	0.03	0.12	0.24	0.77	0.64	0.73	0.12	0.1	0.03	0.05	0.06
Leu	TAG	886	77	127	413	87	203		Y			0.33	0.18	0.03	0.01	0.03	0.42	0.31	0.87	0.81	0.83	0.09	0.09	0.03	0.03	0.02	0.08	0.19	0.02	0.02	0.05
Leu	TAA	731	61	139	263	84	158					0.2	0.22	0.08	0.08	0.04	0.09	0.09	0.07	0.05	0.04	0.2	0.23	0.71	0.43	0.67	0.25	0.16	0.11	0.16	0.1
Tyr	GTA	1 435	91	288	413	263	251		Y	Fe		0.15	0.15	0.11	0.13	0.07	0.18	0.17	0.09	0.1	0.1	0.15	0.28	0.19	0.12	0.11	0.04	0.01	0.06	0.02	0.1
Met	CAT	1 368	125	205	533	214	242					0.02	0.06	0.03	0.02	0.01	0.1	0.05	0.02	0.06	0.05	0.04	0.09	0.13	0.05	0.03	0.01	0.03	0.02	0.03	0.03
Asn	GTT	1 345	96	333	517	157	207					0.14	0.18	0.06	0.06	0.05	0.27	0.27	0.64	0.49	0.58	0.2	0.23	0.06	0.09	0.09	0.05	0.02	0.01	0.04	0.02
Trp	CCA	1 317	90	155	475	295	216		Y			0.16	0.21	0.12	0.06	0.21	0.03	0.06	0.01	0.03	0.02	0.55	0.37	0.66	0.61	0.55	0.03	0.07	0.03	0.04	0.04
SeC	TCA	1 282	93	610	229	90	104					0.01	0.03	0.02	0.03	0.03	0.04	0.02	0.03	0.01	0.04	0.06	0.03	0.14	0.12	0.06	0.16	0.17	0.28	0.23	0.14
Lys	CTT	612	31	157	271	87	62					0.09	0.15	0.07	0.08	0.17	0.04	0.05	0.06	0.07	0.07	0.11	0.12	0.52	0.24	0.39	0.13	0.27	0.08	0.09	0.07
Lys	TTT	342	12	64	172	63	38					0.18	0.13	0.1	0.07	0.19	0	0.09	0.09	0.06	0.09	0.12	0.03	0.12	0.07	0.05	0.06	0.18	0.06	0.15	0.13
Ile	AAT	293	29	108	70	27	29					0.05	0.26	0.28	0.05	0.06	0.17	0.22	0.14	0.09	0.06	0.17	0.08	0.23	0.07	0.39	0	0.09	0.02	0.04	0.03
