# Supplementary material for: The diversity of small non-coding RNAs in the diatom Phaeodactylum tricornutum
Source: BMC Genomics. 2014 Aug 20;15(1):698. doi: 10.1186/1471-2164-15-698 (PMC4247016; doi:10.1186/1471-2164-15-698)
Supplement: Supplementary file 11 — Additional file 11: Table S4: List of primers utilized in the present work. (PDF 39 KB) [file 12864_2014_6681_MOESM11_ESM.pdf]

Additional Table S4

**OLIGOPROBES**	
U2 snRNA	GTACACGGTCAAGGCGGTGAGTG
U6	GCTAATCTTCTCTGTATC
chr_2:29.039-29.123	AAATGTTCGAGAGAGAAATTACAAAGA
Asp-GAC	TCCGACACGGGGAATCGA
Gly-GGC	GCGCCATCCGGGAATCGA
Pro-CCT	GGTCCAAGCCGGGAATCGA
Glu-GAG	CGTCGTATGATAACCACC
**qRT-PCR**	
**Transposons**	
EU432476_chr_18:652040-658588 Fw	GCCACTCAGCCTCCTCCTA
EU432476_chr_18:652040-658588 Rv	TACTGCACAGAAGCACGGTT
EU432476_chr_3:105711-112245 Fw	GCTCCTGGGGAGTCACAAGT
EU432476_chr_3:105711-112245 Rv	AGAGAAGACACGCCATTCGG
EU432478_chr_13:47329-55017 Fw	CTAGTGTGCGTTCCGCTTTG
EU432478_chr_13:47329-55017 Rv	CCCATCATCGTGCGTCCAAT
EU432481_chr_4:346-7718 Fw	TGATACTGGTGCAGACACCG
EU432481_chr_4:346-7718 Rv	AATGAGCGAGGTGGTCTGTG
EU432484_chr_19:625550-631655 Fw	CTTGGGAGGAGCTTCGAGA
EU432484_chr_19:625550-631655 Rv	ATGCCGAAGTGTCTCGTGT
EU432486_chr_14:400706-405979 Fw	ATCACTCTCGCACCGGTAAC
EU432486_chr_14:400706-405979 Rv	CATTGGTGGGTGAAACAGCG
snRNA U2 Fw	TTCGCCTTATTGGCTTTGAT
snRNA U2 Rv	GTACAGGGTCAAGGCGGTGAG
Rps Fw	GTGCAAGAGACCGGACATACC
Rps Rv	CGAAGTCAACCAGGAAACCAA
H4 Fw	TATGAGCTCGCAATCTCACGCACCAG
H4 Rv	CATCCATGGCTGTTGTTTGTTTTCGG
Sh ble 1 Fw	AGGGTACCCATGGCCAAGTT
Sh ble1 Rv	GATGAACAGGGTCACGTCGTC
**5' and 3' RACE PCR snRNA U2**	
3'outer	TTCGCCTTATTGGCTTTGAT
3'inner	ATTTCACTCACCGCCTTGAC
5'outer	GTCAAGGCGGTGAGTGAAAT
5'inner	TCAAAGCCAATAAGGCGAAG
**STEM LOOP qPCR**	
Universal Reverse PCR primer :	AGTGCAGGGTCCGAGGTATTC
**U2 snRNA**	
RT-loop primer U2-3' snRNA	GTCGTATCCAGTGCAGGGTCCGAGGTATTCGCACTGGATACGACGTACAC
Forward U2-3'primer	GTCGTATCCAGTGCAGGGTCCGAGGTATTCGCACTGGATACGACGTACAC
RT-loop primer U2-5' snRNA	GTCGTATCCAGTGCAGGGTCCGAGGTATTCGCACTGGATACGACCTACAC
Forward U2-5'primer	CCGGCGATTGGCTTTGATCTT
RT-loop primersnoRNAR85	
Forward SnoRNAR85	GTGCATTCAAAAGCCCTTACATGT
